# Ex vivo imaging reveals the spatiotemporal control of ovulation

**DOI:** 10.1038/s41556-024-01524-6

**Published:** 2024-10-16

**Authors:** Christopher Thomas, Tabea Lilian Marx, Sarah Mae Penir, Melina Schuh

**Affiliations:** 1 Max Planck Institute for Multidisciplinary Sciences, Göttingen, Germany; 2 IBDM, CNRS - UMR 7288, Aix-Marseille Université, Marseille, France; 3 Promotionskolleg für Medizinstudierende, University Medical Center Göttingen, Göttingen, Germany; 4 Cluster of Excellence ‘Multiscale Bioimaging: from Molecular Machines to Networks of Excitable Cells’, University of Göttingen, Göttingen, Germany

**Keywords:** Cell biology, Confocal microscopy, Multiphoton microscopy

## Abstract

During ovulation, an egg is released from an ovarian follicle, ready for fertilization. Ovulation occurs inside the body, impeding direct studies of its progression. Therefore, the exact mechanisms that control ovulation have remained unclear. Here we devised live imaging methods to study the entire process of ovulation in isolated mouse ovarian follicles. We show that ovulation proceeds through three distinct phases, follicle expansion (I), contraction (II) and rupture (III), culminating in the release of the egg. Follicle expansion is driven by hyaluronic acid secretion and an osmotic gradient-directed fluid influx into the follicle. Then, smooth muscle cells in the outer follicle drive follicle contraction. Follicle rupture begins with stigma formation, followed by the exit of follicular fluid and cumulus cells and the rapid release of the egg. These results establish a mechanistic framework for ovulation, a process of fundamental importance for reproduction.

## Abstract (web summary)

Thomas, Marx et al. devise a live imaging approach to spatiotemporally dissect mouse ovulation ex vivo.

## Main

Immature eggs, called oocytes, are stored in ovarian follicles. Once in every ovarian cycle, an oocyte matures into a fertilizable egg by progressing through the first meiotic division. In time with this, the egg is released from the follicle, in a process termed ovulation. Ovarian follicles consist of an oocyte surrounded by specialized somatic cells called granulosa cells that support the oocyte’s growth and maintain its arrest in meiotic prophase I. In response to follicle-stimulating hormone (FSH), follicles undergo immense growth and remodelling to become pre-ovulatory follicles, which are characterized by a fluid-filled cavity (antrum) next to the oocyte. At this stage, follicles are able to respond to the mid-cycle surge in luteinizing hormone (LH), which triggers oocyte maturation and ovulation^1^.

During ovulation, the follicle undergoes dramatic cellular and extracellular matrix remodelling^2^. These dynamic changes have been challenging to study, given the limited accessibility of this complex process. Much has been learned from genetic approaches^3–5^, animal studies^6,7^ and culture methods for cumulus–oocyte complexes^8^, ovarian tissues^9,10^ and whole ovarian follicles. Follicle culture systems have demonstrated that follicles are functional units that can be used to model many aspects of ovarian biology outside of the animal such as follicle growth^11^, maintenance of oocyte arrest^12^, disease architecture^13^ and ovulation^11,14,15^. They have been instrumental in advancing our understanding of ovarian function.

Live imaging approaches have been key to revealing the dynamic nature of many biological processes. While there has been substantial progress towards live imaging specific features of ovulation in vivo^16,17^, methods to visualize and probe the entire process in high spatiotemporal resolution do not currently exist. There is thus an important gap in our understanding of this essential step in mammalian reproduction.

## Results

### Cultured follicles recapitulate in vivo ovulation

**Fig. 1:**
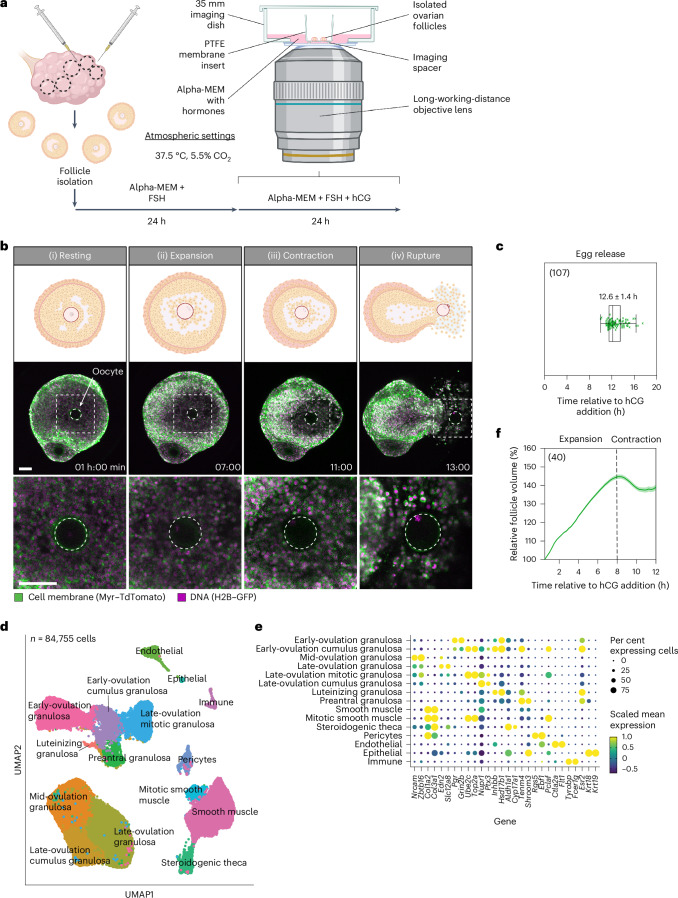
Live imaging reveals follicle expansion and contraction before egg release. **a**, A schematic representation of antral follicle culture and live imaging setup. **b**, Illustrations and representative confocal images demonstrating the phases of ovulation. The dotted white square indicates the region of inset; the dotted white circle indicates the oocyte outline; time is relative to the addition of hCG. Scale bar, 100 μm. **c**, A plot showing the timing of the egg release from the follicle. Total *n* = 107, shown in the top left of the graph, data from 17 independent biological repeats. Box, 25–75%; whiskers, 5–95%; centre, median; centre box, mean. Mean ± s.d. is displayed. **d**, A UMAP visualization illustrating 15 different cell types in the ovulating follicle. Both the ex vivo and the in vivo datasets and all timepoints were included in the analysis. Where cluster names include ‘early’, ‘mid’ or ‘late’ ovulation, this refers to the collection timepoints that are predominantly represented in the cluster (Extended Data Fig. 2b). **e**, The top highly expressed gene in each of the annotated clusters. The size of the dot corresponds to the percentage of cells in a cluster expressing the gene, while the colour indicates the mean scaled expression level of the gene in each cluster. Data in **d** and **e** are representative of three independent biological repeats. **f**, The relative follicle volume (each normalized to value at 0.5 h post hCG) throughout ovulation; *n* = 40, shown in the top left of the graph, data from 6 independent biological repeats. Created with BioRender.com. Source data

**Extended Data Fig. 1:**
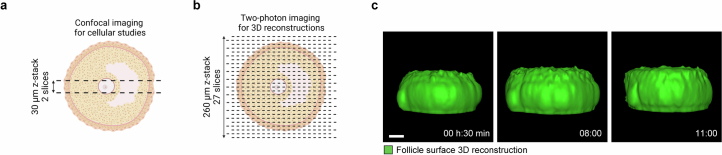
Live imaging reveals follicle expansion and contraction prior to egg release (extended data). (**a**) Schematic representation of strategy for confocal imaging. (**b**) Schematic representation of strategy for 2-photon imaging for 3D reconstructions. (**c**) Representative time course of 3D-reconstructed follicles during ovulation. Time relative to hCG addition; scale bar, 100 μm. Created with BioRender.com.

To investigate the mechanisms that drive ovulation, we adapted mouse follicle culture protocols^10,11,15^ for use with long-term live imaging (Fig. 1a). We triggered ovulation outside of the body by culturing follicles in medium containing the LH analogue human chorionic gonadotrophin (hCG) and FSH. To follow cellular movements, we used transgenic mice expressing a cell membrane (Myr–TdTomato) and a histone (H2B–GFP) marker^18^. We imaged follicles from these mice for up to 24 h after hCG addition (at 10 min intervals), covering the entire process of ovulation. By combining confocal (Fig. 1b, Extended Data Fig. 1a and Supplementary Video 1) and two-photon (Extended Data Fig. 1b,c) microscopy, we visualized ovulation at both the cellular and whole follicle scale. Ovulation was successful in >90% of the follicles imaged. The eggs left the follicle at 12.6 ± 1.4 (mean ± standard deviation (s.d.)) hours post hCG addition (Fig. 1c), consistent with ovulation timing in vivo^19^ and in other studies of ex vivo ovulation^20^.

**Extended Data Fig. 2:**
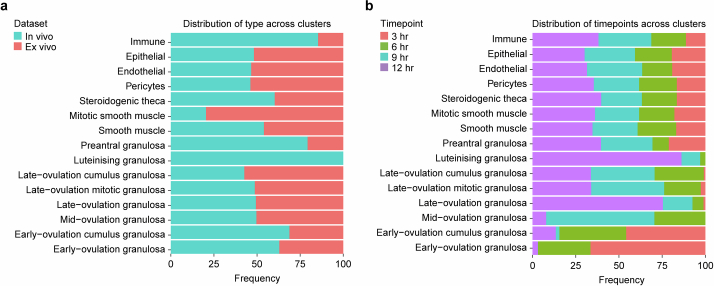
Cluster distribution for single-cell transcriptomic map of murine ovulation. (**a**) Bar plot showing the distribution of cell types between in vivo and ex vivo samples. Most cell types are represented to equivalent extents in both samples, with the exception of luteinising granulosa cells and immune cells which both rely on access to factors derived from outside the follicle during ovulation. (**b**) Bar plot showing the relative contribution of the collection time points to each cluster. All data is representative of 3 independent biological repeats. Source data

To confirm that ovulation in cultured follicles accurately reflects in vivo ovulation, we performed single-cell RNA sequencing (scRNA-seq) on cultured follicles (ex vivo setup) and follicles collected from superovulated C57BL/6J mice (in vivo setup) throughout ovulation. Briefly, we collected follicles from either the ex vivo or in vivo setup at 0, 3, 6, 9 and 12 h post hCG administration. We then prepared single-cell suspensions and subjected the samples to droplet-based 3′-end sequencing (10x Genomics). After removing low-quality cells, we obtained the high-quality transcriptomes of 84,755 cells (Fig. 1d). Unsupervised clustering and assessment of the relative distribution of differential experimental parameters to each cluster showed that cluster assignment reflected physiological differences between cell types (Extended Data Fig. 2a,b). We annotated a total of 15 cell types on the basis of the exclusive and high expression of known marker genes from the literature (Fig. 1e)^21,22^. This scRNA-seq dataset provided the basis for the analysis of cell type-specific transcriptomic changes in the ovulating follicle.

**Extended Data Fig. 3:**
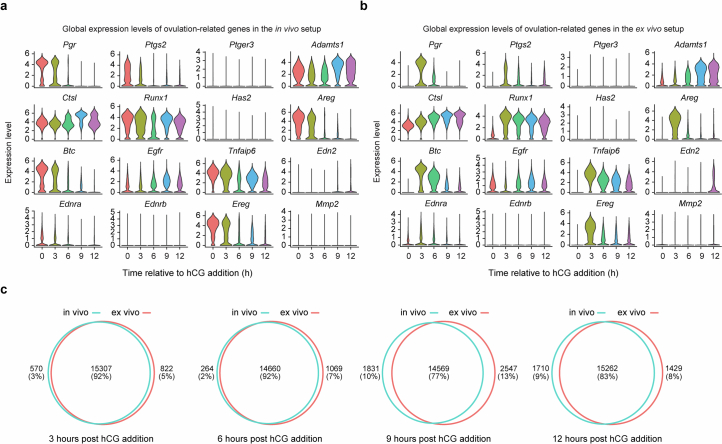
Temporal expression of ovulation-related genes in the in vivo and ex vivo datasets. (**a-b**) Violin plots showing the global changes in expression level of ovulation-related genes across all cell types at 0, 3, 6, 9, and 12 h post hCG administration in both in vivo and ex vivo datasets. At 0 h, prior to hCG administration, the in vivo samples already show expression of genes known to be downstream of LH, such as *Pgr*^23^. This can be explained by previous findings that PMSG injection into mice can cause a spontaneous endogenous LH surge in mice^24^. We therefore excluded the 0 hour time point for all downstream analysis (Fig. 1d, e and further analyses focusing on cell-type specific transcriptomic changes during ovulation). (**c**) Area-proportional Venn diagrams showing the global transcriptomic overlap between the in vivo and ex vivo datasets at 3, 6, 9, and 12 h post hCG administration. Differential gene expression analysis was performed with the LRT test. For the global comparison of the two setups at each time point, the Wald test in DESeq2 v1.32.0 was used. A gene was considered to be upregulated in the dataset if its |avglog2FC | ≥ 0.5 and adjusted p-value < 0.01. All data is representative of 3 independent biological repeats. Source data

To determine whether the temporal expression of key ovulation-related genes is conserved in our follicle culture system, we compared gene expression profiles between the ex vivo and in vivo setups. We observed a strong conservation of the expression timing between ex vivo and in vivo samples collected at 3, 6, 9 and 12 h post hCG administration, but not those collected at 0 h (Extended Data Fig. 3a,b). At 0 h, before hCG administration, the in vivo samples already showed expression of genes known to be downstream of LH, such as *Pgr*^23^. This can be explained by previous findings that pregnant mare serum gonadotropin (PMSG) injection can cause a spontaneous endogenous LH surge in mice^24^. Despite this inconsistency before the onset of ovulation, all timepoints following hCG administration showed consistent expression profiles between the ex vivo and in vivo setups (Extended Data Fig. 3a,b). We therefore proceeded with the dataset from 3, 6, 9 and 12 h, which reflects exclusive changes after hCG administration, for the clustering in Fig. 1d,e and other downstream analyses of cell type-specific transcriptomic changes during ovulation. We then further validated our ex vivo system by comparing global gene expression profiles at individual timepoints through ovulation, demonstrating a large overlap between the in vivo and ex vivo datasets (92% at 3 h, 92% at 6 h, 77% at 9 h and 83% at 12 h post hCG addition (Extended Data Fig. 3c). We conclude that the ex vivo follicle culture system accurately reflects in vivo ovulation. For the rest of this study, we use a combined approach of our ex vivo live imaging system and scRNA-seq dataset to characterize the process of ovulation.

### Ovulation proceeds via three phases

**Extended Data Fig. 4:**
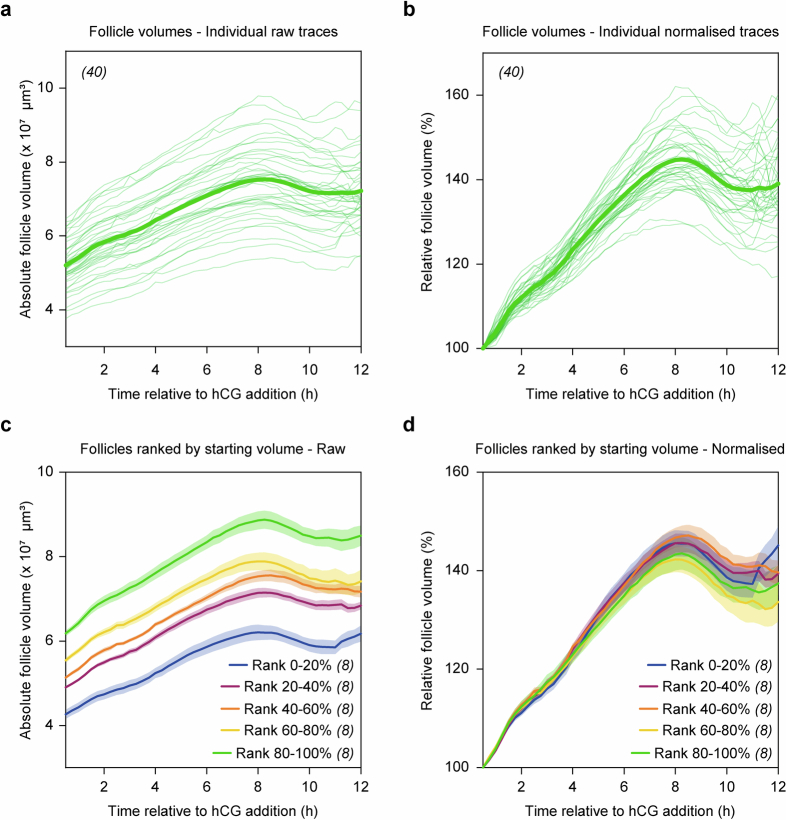
Control follicle volume analysis. (**a**) Graph showing absolute follicle volume, non-normalised. (**b**) Graph showing follicle volume normalised at 0.5 h post hCG addition. (**c**) Graph showing absolute follicle volume of control follicles ordered into 5 groups by their starting volume. (**d**) Graph showing volume of control follicles ordered by their starting volume, normalised at 0.5 h post hCG addition. In panels (a,b), thin traces show raw values from individual follicles; thick traces show the mean. In panels (c,d), thick lines show the mean, lighter shadows show the SEM. Panels (a,b,c,d), contain data from 40 follicles from 6 independent biological repeats. Each group in panels (c,d) contains 8 follicles. Source data

Confocal imaging revealed that, consistently, the follicles first expanded and subsequently contracted upon addition of hCG (Fig. 1b). To quantitatively analyse follicle size changes, we generated three-dimensional (3D) surface reconstructions from two-photon imaging over the entire height of the follicles (Extended Data Fig. 1b,c). During the first 8 h, the follicles expanded in volume (phase I—follicle expansion; Fig. 1b(ii) and 1f and Extended Data Figs. 1c and 4a,b). By 8 h, follicles had expanded to 145 ± 1.1% (mean ± standard error of the mean (s.e.m.)) of the volume at 0.5 h post hCG addition. From 8 h onwards, the follicles contracted (phase II—follicle contraction; Fig. 1b(iii) and 1f and Extended Data Figs. 1c and 4a,b), characterized by a significant decrease in volume. The extent to which the follicles expanded and contracted was independent of the initial volume (Extended Data Fig. 4c,d). Finally, the follicles ruptured, and the eggs were released to the exterior (phase III—rupture; Fig. 1b(iv)). In summary, ovulation proceeds through three discrete and highly synchronous phases: follicle expansion (I), contraction (II) and rupture with egg release (III). Importantly, these phases are consistent with the timing of events during ovulation in vivo^25,26^.

### Hyaluronic acid drives follicle expansion

**Fig. 2:**
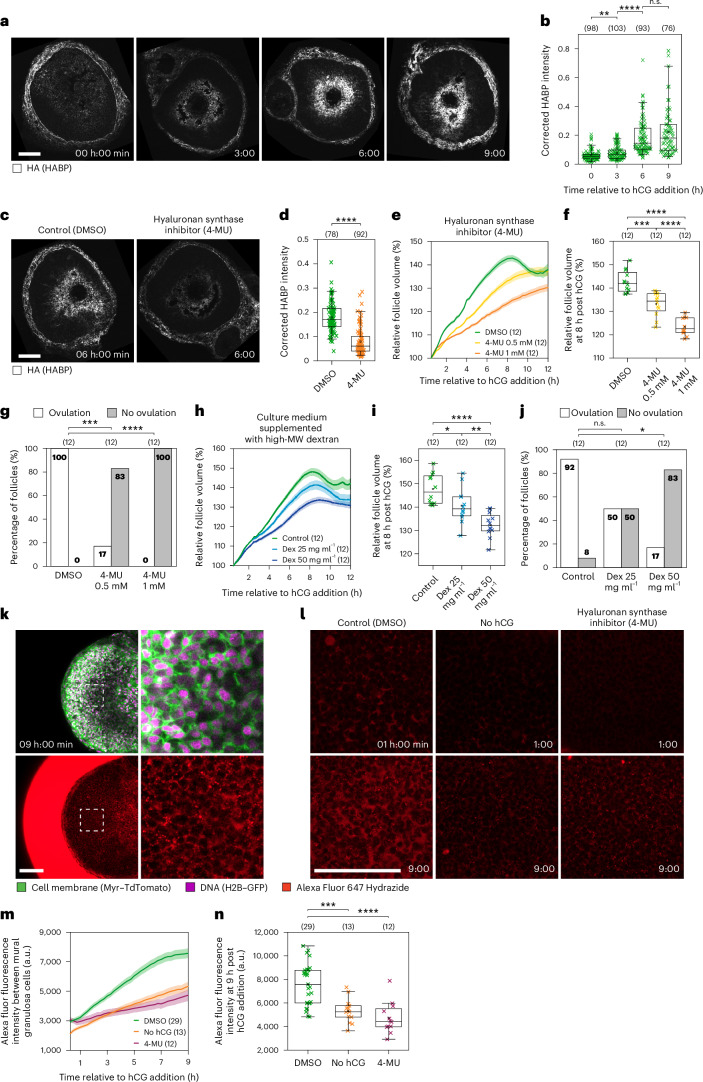
Follicle expansion is driven by HA secretion. **a**, Representative time course of HA (HABP) staining of cryosectioned follicles collected at 0, 3, 6 and 9 h. **b**, The quantification of HABP mean fluorescence intensity normalized to Hoechst intensity. Total *n* = 370; individual groups: 0 h *n* = 98, 3 h *n* = 103, 6 h *n* = 93, 9 h *n* = 76, shown above the graph. *P* values: 0 h versus 3 h = 0.001558902, 3 h versus 6 h = 1.23167 × 10^−14^, 6 h versus 9 h = 0.095707099. **c**, HA staining of DMSO- and 4-MU-treated follicles at 6 h. **d**, The quantification of HA mean fluorescence intensity in DMSO- and 4-MU-treated follicles. Total *n* = 170; individual groups: DMSO *n* = 78, 4-MU *n* = 92, shown above the graph. *P* = 7.0863 × 10^−21^. **e**, The relative volume in DMSO- and 4-MU-treated follicles. **f**, A statistical comparison of the volumes in DMSO- and 4-MU-treated follicles at 8 h. *P* values: control versus 0.5 mM = 1.02 × 10^−4^, control versus 1 mM = 1.51 × 10^−10^, 0.5 mM versus 1 mM = 2.21 × 10^−5^. **g**, Ovulation rates in DMSO- and 4-MU-treated follicles. *P* values: control versus 0.5 mM = 0.00001, control versus 1 mM = 0.000194. In **e**–**g**, total *n* = 36; individual groups: DMSO *n* = 12, 0.5 mM 4-MU *n* = 12, 1 mM 4-MU *n* = 12, shown above the graphs in **f** and **g**. **h**, The relative volume in control and dextran (Dex)-treated follicles. **i**, A statistical comparison of volumes in control and dextran-treated follicles at 8 h. *P* values: control versus 25 mg ml^−1^ = 1.98 × 10^−2^, control versus 50 mg ml^−1^ = 1.96 × 10^−6^, 25 mg ml^−1^ versus 50 mg ml^−1^ = 4.97 × 10^−3^. **j**, Ovulation rates in control and dextran-treated follicles. *P* values: control versus 25 mg ml^−1^ = 0.160586, control versus 50 mg ml^−1^ = 0.001048. In **h**–**j**, total *n* = 36; individual groups: control *n* = 12, 25 mg ml^−1^ dextran *n* = 12, 50 mg ml^−1^ dextran *n* = 12, shown above the graphs in **i** and **j**. **k**, Representative confocal images of AF647 Hydrazide uptake by the follicle at 9 h. The dotted white square indicates the region of inset. **l**, Representative confocal images of AF647 Hydrazide uptake by DMSO-, no hCG- and 4-MU-treated follicles at 1 and 9 h. **m**, The AF647 fluorescence intensity in DMSO-, no hCG- and 4-MU-treated follicles. **n**, A statistical comparison of AF647 fluorescence intensity in DMSO-, no hCG- and 4-MU-treated follicles at 9 h. *P* values: control versus no hCG = 1.24 × 10^−4^, control versus 4-MU = 1.15 × 10^−5^. In **m**,**n**, total *n* = 54; individual groups: DMSO *n* = 29, no hCG *n* = 13, 4-MU *n* = 12, shown above the graph in **n**. Data in **a**–**n** are representative of two independent biological repeats. In **e**, **h** and **m**, the thick lines show the mean, and the lighter shadows show the s.e.m. In **b**, **d**, **f**, **i** and **n**: ****P < 0.0001, ***P < 0. 001, **P < 0.01, n.s. non-significant, two-sided unpaired *t*-test; box, 25–75%; whiskers, 5–95%; centre, median; centre box, mean. In **g** and **j**: chi-square test (two-sided) with Yates’s correction. All times are relative to hCG addition. All scale bars, 100 μm. Source data

**Extended Data Fig. 5:**
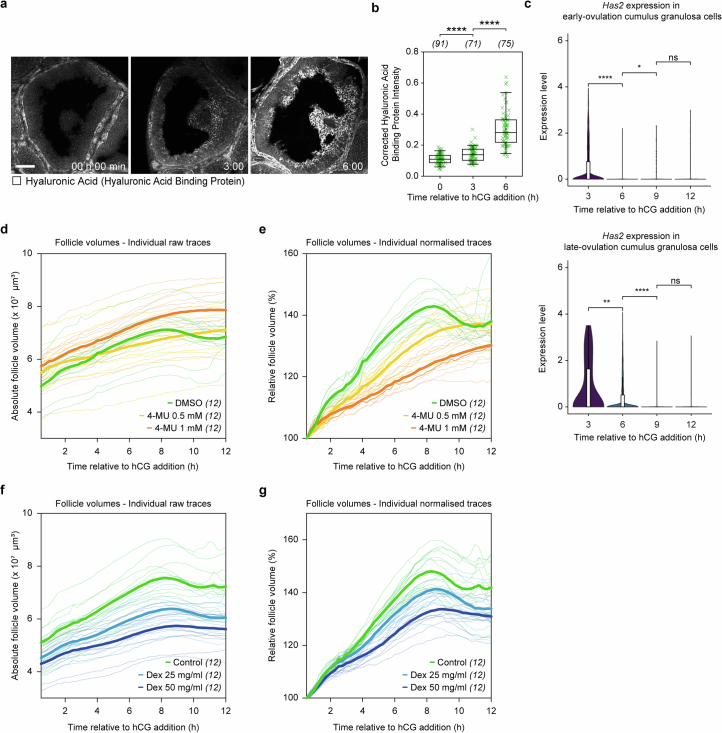
Follicle expansion is driven by hyaluronic acid secretion (extended data). (**a**) Representative image of hyaluronic acid (HA) using HA-Binding Protein (HABP) staining of cryosectioned whole ovaries collected at 0, 3, and 6 h post hCG addition. Scale bar, 100 μm. (**b**) Quantification of HABP mean fluorescence intensity in the inner follicle normalised to Hoechst intensity. 0 h *n* = 91, 3 h *n* = 71, 6 h *n* = 75. Box = 25-75%, whiskers = 5-95%, centre = median, centre box = mean. P values: 0v3h = 2.62364E-06, 3v6h = 3.87849E-22. (**c**) Violin plot showing *Has2* expression in cumulus granulosa cells during ovulation. Box = 25-75%, whiskers = min and max, centre = median. P values = Early ovulation cumulus granulosa cells 3v6 = <2.22E-16, 6v9 = 0.017, 9v12 = 0.16; Late ovulation cumulus granulosa cells 3v6 = 0.0081, 6v9 = <2.22E-16, 9v12 = 0.13. (**d**) Graph showing absolute volume, non-normalised, in DMSO and 4-MU-treated follicles. (**e**) Graph showing volume normalised at 0.5 h post hCG addition in DMSO and 4-MU-treated follicles. Panels (d,e): total *n* = 36; individual groups: DMSO *n* = 12, 0.5 mM 4-MU *n* = 12, 1 mM 4-MU *n* = 12. (**f**) Graph showing absolute volume, non-normalised, in DMSO and dextran-treated follicles. (**g**) Graph showing volume normalised at 0.5 h post hCG addition in DMSO and dextran-treated follicles. Panels (f,g): total *n* = 36; individual groups: DMSO *n* = 12, 25 mg/ml dextran *n* = 12, 50 mg/ml dextran *n* = 12. Data in panels (a,b,d,e,f,g) representative of 2 independent biological repeats. Data in (c) representative of 3 independent biological repeats. In panels (b,c): ****P < 0.0001, ***P < 0. 001, **P < 0.01, n.s. non-significant, two-sided unpaired t-test. In panels (d,e,f,g): thin traces show raw values from individual follicles; thick traces show the mean. Source data

We next investigated the mechanisms driving the phases of ovulation, starting with follicle expansion. Expansion of a tissue is often driven by cell divisions. However, we observed few dividing cells during follicle expansion (Supplementary Video 2). Given the rapid rate of follicle expansion, we instead asked whether it is driven by extracellular matrix remodelling. Several recent studies have shown that hyaluronic acid (HA) secretion can generate rapid tissue remodelling by inducing localized hydrostatic pressure^27–29^. It is well established that the cumulus cells of the inner follicle secrete HA in response to the LH surge^8,30,31^. To confirm and further characterize the dynamics of HA secretion in follicles during ovulation, we stained for HA in cryosectioned follicles collected at 0, 3, 6 and 9 h post hCG addition. HA was enriched in the antrum surrounding the oocyte and increased significantly during the first 6 h following hCG addition (132% at 3 h and 307% at 6 h; Fig. 2a,b), consistent with HA secretion patterns during in vivo ovulation (127% at 3 h and 274% at 6 h; Extended Data Fig. 5a,b). In line with this, hyaluronic acid synthase 2 (*Has2*) messenger RNA was strongly upregulated in cumulus cells at 3 h post hCG addition (Extended Data Fig. 5c). We hypothesized that an increase in HA could generate an osmotic gradient towards the inside of the follicle, leading to fluid influx and follicle expansion. To test this hypothesis, we treated follicles with an inhibitor of hyaluronic acid synthase (4-MU)^32,33^. This significantly reduced the amount of HA secreted in response to hCG addition (Fig. 2c,d). Furthermore, follicle expansion was significantly reduced by 8 h post hCG addition in a dose-dependent manner (dimethyl sulfoxide (DMSO), 143% of volume at 0.5 h; 0.5 mM 4-MU, 133%; 1 mM 4-MU, 123%; Fig. 2e,f and Extended Data Fig. 5d,e), and ovulation was blocked (100% ovulation in DMSO, 17% in 0.5 mM 4-MU and 0% in 1 mM 4-MU; Fig. 2g). We thus conclude that HA secretion drives follicle expansion and is essential for ovulation.

To directly test that follicle expansion is driven by an osmotic gradient between the inside and outside of the follicle, we supplemented the culture medium with high-molecular-weight dextran (1,500–2,800 kDa). This dextran cannot diffuse into the follicle as it is substantially larger than the 100 kDa filter of the blood–follicle barrier^34^. Validating our hypothesis, dextran supplementation caused a significant and dose-dependent decrease in follicle expansion (Fig. 2h,I and Extended Data Fig. 5f,g) and ovulation rate (92% ovulation in control, 50% in 25 mg ml^−1^ dextran, 17% in 50 mg ml^−1^ dextran; Fig. 2j). To directly test whether fluid influx occurs during follicle expansion, we added a low-molecular-weight dye to our culture medium and quantified its movement into the follicles during ovulation (Fig. 2k). In control follicles, we observed a marked increase in dye fluorescence intensity between mural granulosa cells by 9 h post hCG addition. The increase in fluorescence intensity was significantly lower when HA secretion was blocked or when follicles were not stimulated to ovulate (Fig. 2l–n). Taken together, these results confirm that fluid influx into the follicle contributes to follicle expansion.

### Follicle contraction is mediated by smooth muscle cells

Follicle contraction initiates 8 h after hCG addition (Fig. 1f). Given that contraction followed a prolonged period of follicle expansion, we first asked whether expansion was essential for contraction. To address this, we examined follicles in which expansion was disrupted by the addition of high-molecular-weight dextran. The dextran-treated follicles expanded significantly less but still contracted with normal timing (Fig. 2h), suggesting that follicles do not need to fully expand in order to contract.

**Fig. 3:**
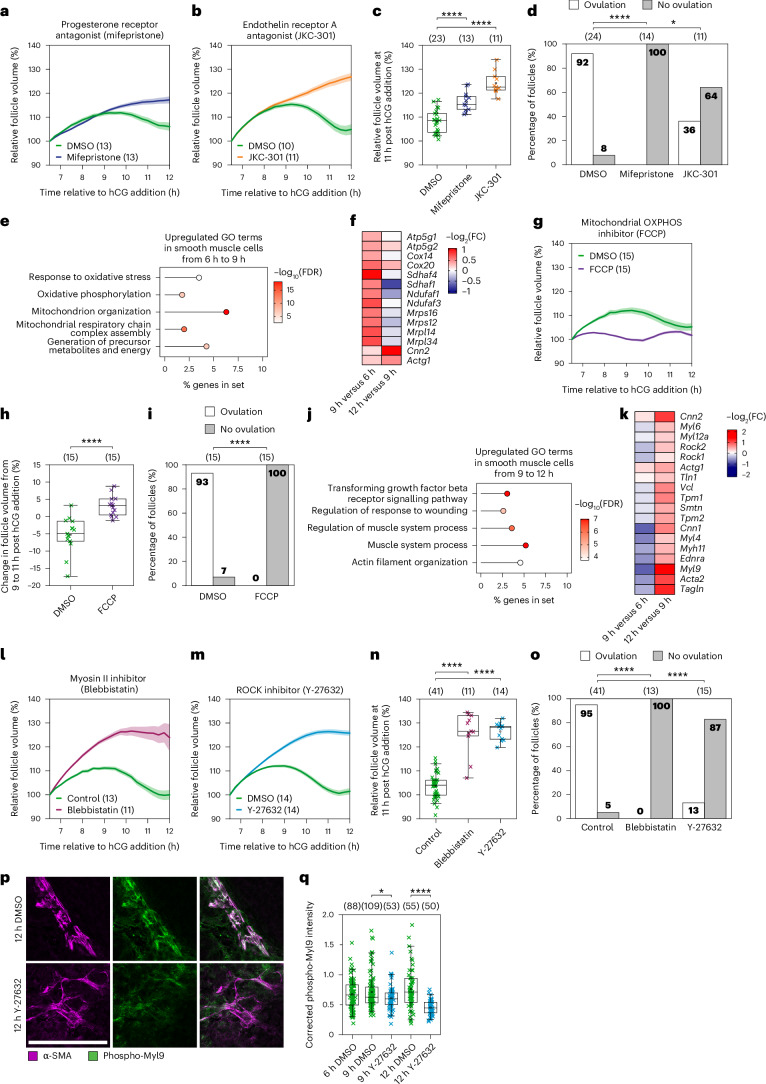
Follicle contraction is mediated by smooth muscle cells. **a**, The relative volume of DMSO- and mifepristone-treated follicles. Total *n* = 26; individual groups: DMSO *n* = 13, mifepristone *n* = 13. **b**, The relative volume of DMSO- and JKC-301-treated follicles. Total *n* = 21; individual groups: DMSO *n* = 10, JKC-301 *n* = 11. **c**, The relative volume at 11 h of DMSO-, mifepristone- and JKC-301-treated follicles. *P* values: control versus mifepristone = 6.13 × 10^−6^, control versus JKC-301 = 1.29 × 10^−10^. **d**, The ovulation rate in DMSO-, mifepristone- and JKC-301-treated follicles. *P* values: control versus mifepristone = 0.000027, control versus JKC-301 = 0.008636. In **c**,**d**, total *n* = 47; individual groups: DMSO *n* = 23, mifepristone *n* = 13, JKC-301 *n* = 11, as shown above the graphs. **e**, Upregulated GO terms in smooth muscle cells from 6 to 9 h. FDR, false discovery rate. **f**, A condensed heatmap of mitochondrial respiratory chain gene expression in smooth muscle cells during follicle contraction. **g**, The relative volume of DMSO- and FCCP-treated follicles. DMSO *n* = 15, FCCP *n* = 15. OXPHOS, oxidative phosphorylation. **h**, The change in relative volume between 9 h and 11 h of DMSO- and FCCP-treated follicles. *P* = 6.56 × 10^−6^. **i**, The ovulation rate in DMSO- and FCCP-treated follicles. *P* = 0.00001. In **h**,**i**, total *n* = 30; individual groups: DMSO *n* = 15, FCCP *n* = 15, as shown above the graphs. **j**, Upregulated GO terms in smooth muscle cells from 9 to 12 h. FDR, false discovery rate. **k**, A heatmap of actomyosin contraction gene expression in smooth muscle cells during follicle contraction. **l**, The relative volume of control ((+) blebbistatin inactive enantiomer) and blebbistatin ((−) blebbistatin active enantiomer)-treated follicles. Total *n* = 24; individual groups: Control *n* = 13, blebbistatin *n* = 11. **m**, The relative volume of DMSO- and Y-27632-treated follicles. Total *n* = 28; individual groups: DMSO *n* = 14, Y-27632 *n* = 14. **n**, The relative volume at 11 h of control and blebbistatin- and Y-27632-treated follicles. *P* values: control versus blebbistatin = 1.98 × 10^−14^, control versus Y-27632 = 7.94 × 10^−21^. Total *n* = 66; individual groups: Control *n* = 41, blebbistatin *n* = 11, Y-27632 *n* = 14, as shown above the graph. **o**, The ovulation rate in control and blebbistatin- and Y-27632-treated follicles. *P* values: control versus blebbistatin = 0, control versus Y-27632 = 0.000016. Total *n* = 69; individual groups: DMSO *n* = 41, blebbistatin *n* = 13, Y-27632 *n* = 15, as shown above the graph. **p**, Representative images of α-SMA and phospho-Myl9 staining in cryosectioned follicles collected at 12 h ± Y-27632. Scale bar, 100 μm. **q**, Quantification of phospho-Myl9 mean fluorescence intensity in the outer follicle normalized to the mean fluorescence intensity of α-SMA at 6, 9 and 12 h ± Y-27632. 6 h DMSO *n* = 88, 9 h DMSO *n* = 109, 9 h Y-27632 *n* = 53, 12 h DMSO *n* = 55, 12 h Y-27632 *n* = 50, as shown above the graph. *P* values: 9 h control versus Y-27632 = 0.03368, 12 h control versus Y-27632 = 2.37 × 10^−8^. Data in **a**–**d**, **g**–**i** and **l**–**q** are representative of two independent biological repeats. Data in **e**, **f**, **j** and **k** are representative of three independent biological repeats. In **a**, **b**, **g**, **l** and **m**, the thick lines show the mean, and the lighter shadows show the s.e.m. In **c**, **h**, **n** and **q**: ****P < 0.0001, ***P < 0. 001, **P < 0.01, n.s. non-significant, two-sided unpaired *t*-test; box, 25–75%; whiskers, 5–95%; centre, median; centre box, mean. In **d**, **i** and **o**: chi-square test (two-sided) with Yates’s correction. All times are relative to hCG addition. Source data

**Extended Data Fig. 6:**
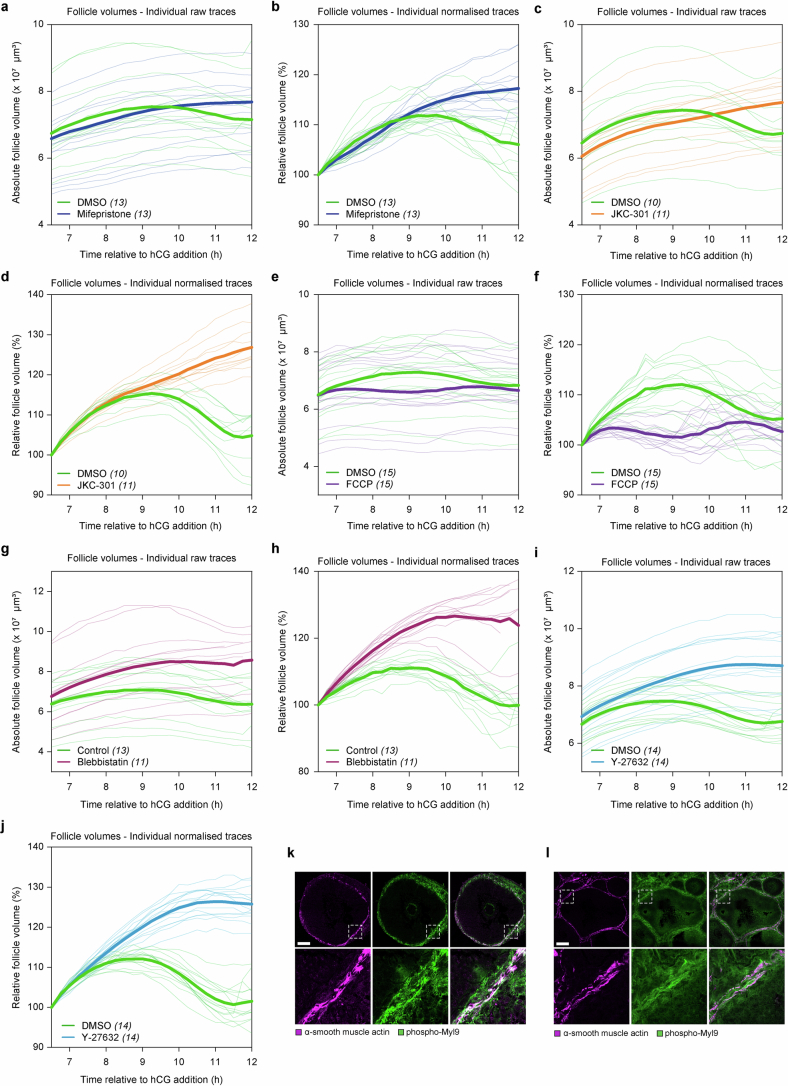
Follicle contraction is mediated by smooth muscle cells (extended data). (**a**) Graph showing absolute volume, non-normalised, in DMSO and mifepristone-treated follicles. (**b**) Graph showing volume normalised at 6.5 h post hCG addition in DMSO and mifepristone-treated follicles. Panels (a,b): total *n* = 26; individual groups: DMSO *n* = 13, mifepristone *n* = 13. (**c**) Graph showing absolute volume, non-normalised, in DMSO and JKC-301-treated follicles. (**d**) Graph showing volume normalised at 6.5 h post hCG addition in DMSO and JKC-301-treated follicles. Panels (c,d): total *n* = 21; individual groups: DMSO *n* = 10, JKC-301 *n* = 11. (**e**) Graph showing absolute volume, non-normalised, in DMSO and FCCP-treated follicles. (**f**) Graph showing volume normalised at 6.5 h post hCG addition in DMSO and FCCP-treated follicles. Panels (e,f): total *n* = 30; individual groups: DMSO *n* = 15, FCCP *n* = 15. (**g**) Graph showing absolute volume, non-normalised, in control and blebbistatin-treated follicles. (**h**) Graph showing volume normalised at 6.5 h post hCG addition in control and blebbistatin-treated follicles. Panels (g,h): total *n* = 24; individual groups: DMSO *n* = 13, blebbistatin *n* = 11. (**i**) Graph showing absolute volume, non-normalised, in DMSO and Y-27632-treated follicles. (**j**) Graph showing volume normalised at 6.5 h post hCG addition in DMSO and Y-27632-treated follicles. Panels (i,j): total *n* = 28; individual groups: DMSO *n* = 14, Y-27632 *n* = 14. (**k**) Representative whole follicle image of α-SMA and phospho-Myl9 staining of cryosectioned follicles collected at 6, 9, and 12 h post hCG addition. Here, the 9 hour timepoint is shown. (**l**) Representative image of α-SMA and phospho-Myl9 staining of cryosectioned whole ovaries collected at 6, 9, and 12 h post hCG addition. Here, the 12 h timepoint is shown. Data in panels (a,b,c,d,e,f,g,h,i,j,k,l) representative of 2 independent biological repeats. In panels (a,b,c,d,e,f,g,h,i,j): thin traces show raw values from individual follicles; thick traces show the mean. Source data

We hypothesized that follicle contraction is initiated by signalling pathways downstream of hCG. Progesterone receptor expression in granulosa cells peaks 4–8 h after hCG administration in superovulated mice^35^. In addition, blocking progesterone signalling prevents ovulation in mice^36,37^. To test whether progesterone signalling is important for follicle contraction, we treated follicles with a progesterone receptor antagonist (mifepristone). Mifepristone significantly reduced follicle contraction (Fig. 3a,c and Extended Data Fig. 6a,b) and blocked ovulation (92% ovulation in DMSO, 0% in mifepristone; Fig. 3d).

One of the downstream targets upregulated by progesterone signalling is endothelin 2 (ET-2), which is expressed in granulosa cells during ovulation^3,38^ and has been shown to induce contractions in rat ovarian tissue strips^4^. Furthermore, mice with a granulosa cell-specific knockout of ET-2 ovulate significantly fewer oocytes^39^. To test whether ET-2 signalling is involved in follicle contraction, we treated follicles with an endothelin receptor A (EDNR-A) antagonist (JKC-301). In these follicles, contraction was significantly reduced (Fig. 3b,c and Extended Data Fig. 6c,d) and only 36% of the follicles successfully ovulated (Fig. 3d). Together, these results show that progesterone and endothelin signalling drive follicle contraction and are essential for ovulation.

**Extended Data Fig. 7:**
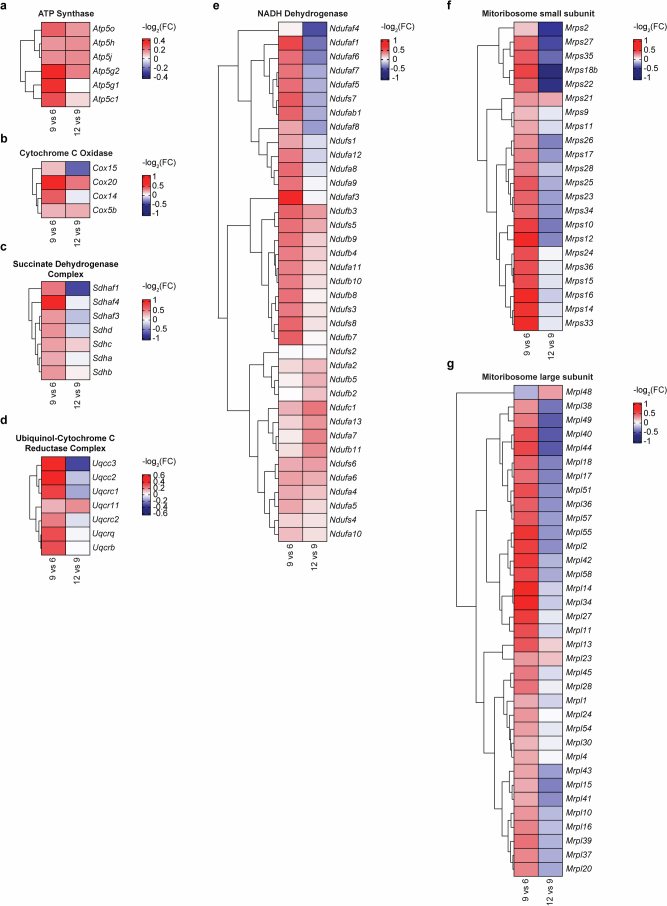
Genes related to the mitochondrial respiratory chain are upregulated in smooth muscle cells at the start of follicle contraction. Expanded heatmap of mitochondrial respiratory chain gene expression in smooth muscle cells during follicle contraction including genes for components of (**a**) ATP synthase, (**b**) Cytochrome C Oxidase, (**c**) Succinate dehydrogenase complex, (**d**) Ubiquinol-Cytochrome C Reductase Complex, (**e**) NADH dehydrogenase, (**f**) Mitoribosome small subunit and (**g**) mitoribosome large subunit. All data is representative of 3 independent biological repeats. Source data

Endothelin has been hypothesized to stimulate the contraction of smooth muscle cells, which has been observed by electron microscopy and immunostaining in the outer layer of antral follicles^3,38,40–44^. Consistent with this, we saw high expression of EDNR-A mRNA (*Ednra*) in smooth muscle cells during ovulation in our scRNA-seq dataset. However, direct experimental evidence for the role of smooth muscle cells in contraction and details of the underlying molecular pathways remain elusive. To address this, we analysed smooth muscle cell-specific transcriptomic changes during the contractile period. Between 6 h and 9 h, we observed an enrichment of Gene Ontology (GO) terms related to mitochondrion organization and mitochondrial respiratory chain complex assembly (Fig. 3e). In addition, there was a striking enrichment of upregulated differentially expressed genes related to the mitochondrial respiratory chain (Fig. 3f and Extended Data Fig. 7). This is consistent with smooth muscle cell contraction being dependent on high levels of ATP production by mitochondrial respiration^45^. To test the role of mitochondrial function in follicle contraction, we treated follicles with the mitochondrial inhibitor carbonyl cyanide *p*-trifluoromethoxyphenylhydrazone (FCCP)^46^. FCCP-treated follicles increase in volume during the normal contractile window between 9 h and 11 h post hCG addition (Fig. 3g,h and Extended Data Fig. 6e,f) and fail to ovulate (Fig. 3i). Taken together, these data establish that mitochondrial energy production is essential for early follicle contraction and ovulation.

We next examined transcriptional changes in smooth muscle cells between 9 h and 12 h post hCG addition and observed an enrichment of GO terms related to muscle system processes and actin filament organization (Fig. 3j). In addition, there was a strong enrichment of upregulated differentially expressed genes related to smooth muscle actomyosin contractility, of which *Acta2* (α-SMA), *Cnn2* (calponin 2), *Myl9* (myosin light chain 9) and *Tagln* (transgelin, SM22) showed the highest fold change (Fig. 3k). Actomyosin-mediated cell contractility results from interactions between the F-actin network and myosin II^47^. To test the role of myosin II in follicle contraction, follicles were treated with blebbistatin, an inhibitor of myosin II. In these follicles, contraction was significantly reduced at 11 h post hCG addition (Fig. 3l,n and Extended Data Fig. 6g,h) and all follicles failed to ovulate (95% ovulation in control, 0% in blebbistatin; Fig. 3o). Contraction of the F-actin network by myosin II is controlled by phosphorylation of Myl9 on Ser19^48^. Interestingly, Rho-associated kinase (ROCK), whose corresponding genes *Rock1* and *Rock2* are upregulated in smooth muscle cells between 9 h and 12 h post hCG addition (Fig. 3k), has been shown to regulate the level of Myl9 phosphorylation through direct inhibition of myosin light chain phosphatase^49^. To test the contribution of ROCK to follicle contraction, we treated follicles with Y-27632, a ROCK inhibitor. Strikingly, follicles treated with the ROCK inhibitor had significantly reduced contraction at 11 h post hCG addition (Fig. 3m,n and Extended Data Fig. 6i,j) and only 13% of follicles ovulated (Fig. 3o), suggesting that it plays a major role in follicle contraction.

Interestingly, we only observed an enrichment of genes related to actomyosin contractility between 9 h and 12 h post hCG addition, once follicle contraction is already underway. This upregulation may be in response to contraction in order to sustain the contractile state of the follicle. To confirm that the actomyosin contractile machinery is present in smooth muscle cells throughout the contractile period, we stained for α-SMA and phospho-Myl9 in cryosectioned follicles and ovaries collected at 6, 9 and 12 h post hCG addition. We observed a strong occupancy of phospho-Myl9 on α-SMA filaments in smooth muscle cells around the follicle throughout the contractile period (Fig. 3p,q and Extended Data Fig. 6k,l). Furthermore, this occupancy was significantly reduced in follicles treated with Y-27632 (Fig. 3p,q). Taken together, these data suggest that follicle contraction is mediated by ROCK- and myosin II-dependent contraction of smooth muscle cells in the outer layer of the follicle.

### Rupture formation and egg release

**Extended Data Fig. 8:**
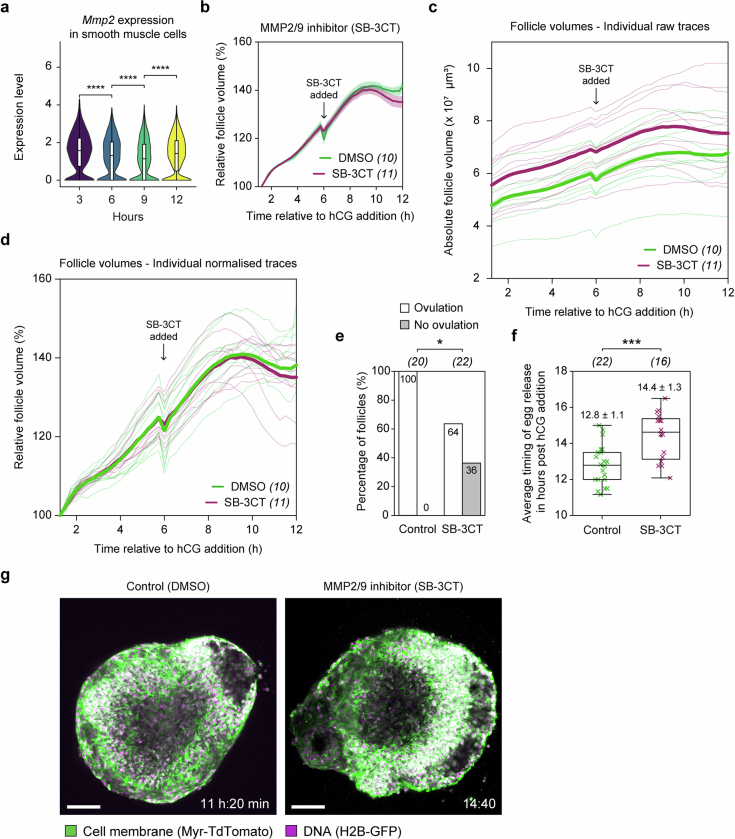
MMP activity is required for follicle rupture but not expansion or contraction. (**a**) Violin plot showing *Mmp2* expression in smooth muscle cells during ovulation. Box = 25-75%, whiskers = min and max, centre = median. P values = 3v6 = 1.5E-15, 6v9 = 6.7E-08, 9v12 = <2.22E-16. (**b**) Relative volume in control (DMSO) and SB-3CT-treated follicles. (**c**) Graph showing absolute volume, non-normalised, in DMSO and SB-3CT-treated follicles. (**d**) Graph showing volume normalised at 1.5 h post hCG addition in DMSO and SB-3CT-treated follicles. Panels (b,c,d): total *n* = 21; individual groups: DMSO *n* = 10, SB-3CT *n* = 11. (**e**) Quantification of ovulation rate in DMSO and SB-3CT-treated follicles. Data from 42 follicles; individual groups: DMSO *n* = 20, SB-3CT *n* = 22. P = 0.009216. (**f**) Quantification of the average timing of egg release in DMSO and SB-3CT-treated follicles. Data from 38 follicles; individual groups: DMSO *n* = 22, SB-3CT *n* = 16. Box = 25-75%, whiskers = 5-95%, centre = median, centre box = mean. Mean ± s.d. is displayed. P = 2.53E-04. (**g**) Representative confocal images of rupture formation in DMSO and SB-3CT-treated follicles. Data in (a) is representative of 3 independent biological repeats. Data in panels (b,c,d) is representative of 2 independent biological repeats. Data in panels (e,f) is representative of 4 independent biological repeats. In panels (a,f): ****P < 0.0001, ***P < 0. 001, **P < 0.01, n.s. non-significant, two-sided unpaired t-test. In panel (e), chi-square test (two-sided) with Yates’s correction. In panel (a): thick lines show the mean, lighter shadows show the SEM. In panels (c,d): thin traces show raw values from individual follicles; thick traces show the mean. Source data

In the final stage of ovulation, the egg needs to be released from the follicle. The outer wall of the follicle first weakens and then ruptures^50,51^. Follicle rupture is facilitated by local tissue weakening through the action of proteases^52^. Consistent with this, we observed strong expression of matrix metalloprotease 2 (*Mmp2*) in smooth muscle cells throughout ovulation in our scRNA-seq dataset (Extended Data Fig. 8a). To test the role of MMP2 activity in rupture formation and ovulation, follicles were treated with SB-3CT, an inhibitor of MMP2/9 activity. In these follicles, expansion and contraction proceeded normally (Extended Data Fig. 8b–d), but only 64% of the follicles ovulated (Extended Data Fig. 8e). Of these SB-3CT-treated follicles that ovulated, the time of egg release was significantly later (14.4 ± 1.3 h, mean ± s.d.) than in control follicles (12.8 ± 1.1 h, mean ± s.d.; Extended Data Fig. 8f). Confocal imaging revealed that control follicles distended outwards in the thinnest region of the follicle wall during the final hour before egg release, eventually forming a localized rupture site. In contrast, SB-3CT-treated follicles were unable to form a rupture site efficiently. Instead, the follicle wall remained intact and precluded egg release. Taken together, these data confirm that MMP2 activity is required for follicle rupture but not for expansion or contraction.

**Fig. 4:**
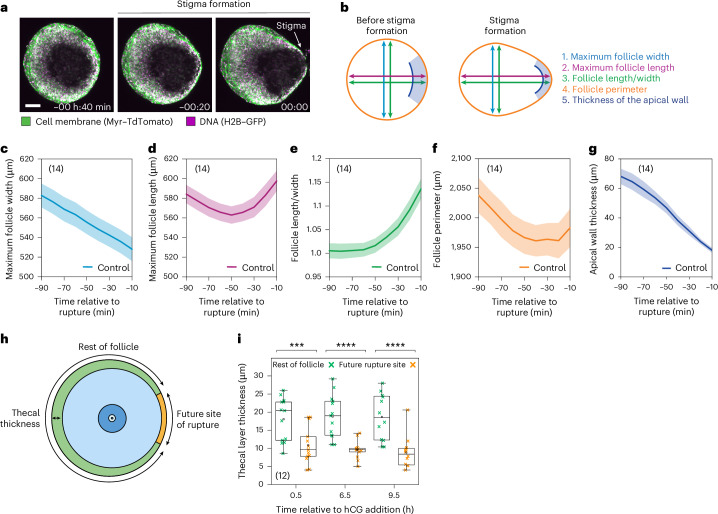
Stigma formation precedes follicle rupture. **a**, Representative time course confocal images of stigma formation. Time is relative to the rupture of the follicle wall. Scale bar, 100 μm. **b**, A schematic explaining measurements made in **c**–**g**. **c**, The mean maximum follicle width during stigma formation. **d**, The mean maximum follicle length during stigma formation. **e**, The mean follicle length/width ratio during stigma formation. **f**, The mean maximum follicle perimeter during stigma formation. **g**, The mean apical wall thickness during stigma formation. **h**, A schematic illustration explaining how thickness measurements were made in **i**. **i**, The theca layer thickness on future rupture site and rest of the follicle at 0.5, 6.5 and 9.5 h after hCG addition. *P* values: 0 h = 2.93 × 10^−3^, 6 h = 2.77 × 10^−5^, 9 h = 2.41 × 10^−4^. Data in **a** and **c**–**g** are representative of 14 control follicles, as shown in the top left of the graphs over 2 independent biological repeats. In **c**–**g**, the thick lines show the mean, and the lighter shadows show the s.e.m. Data in **i** are from 12 follicles, as shown in the bottom left of the graph over 4 independent biological repeats. In **i**: ***P < 0. 001; ****P < 0.0001, two-sided unpaired *t*-test; box, 25–75%; whiskers, 5–95%; centre, median; centre box, mean. Created with BioRender.com. Source data

The distension formed in the final hour of ovulation is termed the stigma (Fig. 4a) and has previously been recorded at low resolution in multiple living mammals, including humans^53,54^. However, the timing of stigma formation and its relationship to egg release has remained unclear. To address this, we quantified how follicle shape changes before rupture. Specifically, we measured follicle length (diameter parallel to rupture), follicle width (diameter perpendicular to rupture) and follicle perimeter (Fig. 4b). While the follicle width decreased steadily during contraction up until the point of rupture (Fig. 4c,e), the follicle length first decreased, and then increased rapidly during the final 50 min as the follicle distended (Fig. 4d,e). This distension was associated with a late increase in follicle perimeter (Fig. 4f). The wall thickness at the future rupture site decreased rapidly during stigma formation, and often comprised only a single cell layer just before rupture (Fig. 4a,g).

We observed that the stigma typically formed in the thinnest region of the outermost follicle layer (thecal layer; Fig. 4a). To quantify this, we measured the thickness of the thecal layer at the future rupture site and around the rest of the follicle throughout ovulation (Fig. 4h). This analysis revealed that, just after stimulation by hCG, the thecal layer at the future rupture site was already significantly thinner than around the rest of the follicle (Fig. 4i, 0.5 h), potentially reflecting a pre-determined polarity in the follicle architecture.

**Fig. 5:**
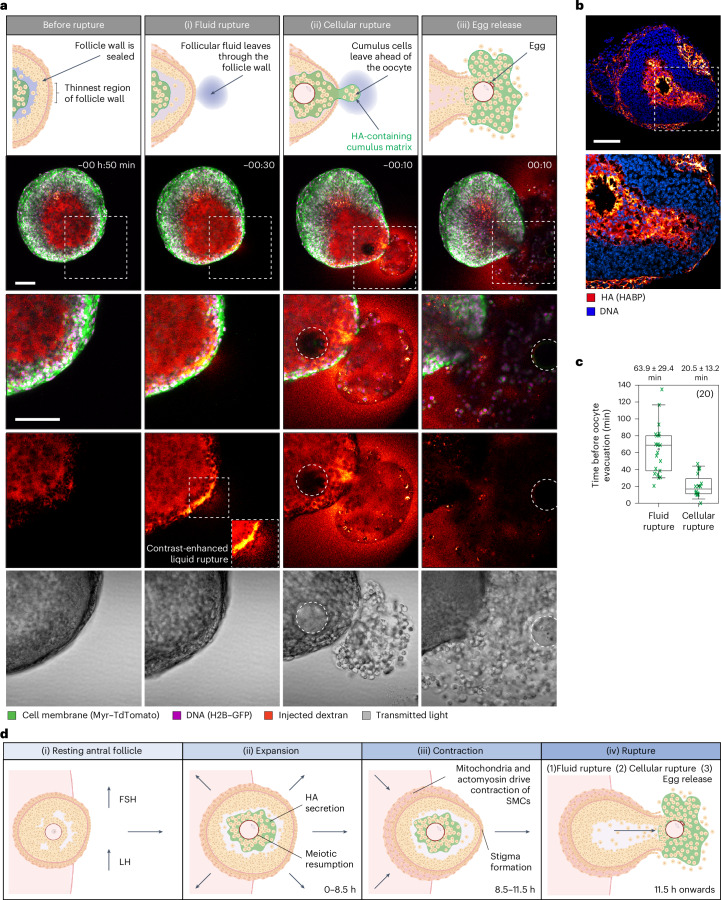
Follicle rupture is a three-step process. **a**, Illustrations and representative confocal images of fluid rupture, cellular rupture and egg release. Inset: contrast-enhanced injected dextran channel to better visualize fluid rupture. The dotted white square indicates the region of inset; the dotted white circle indicates the oocyte outline; time is relative to the egg release. Scale bar, 100 μm. **b**, Immunofluorescence images of cryosectioned follicle collected during stigma formation at 12 h after hCG and stained for HA using HABP. Scale bar, 100 μm. The images are representative of two independent biological repeats. **c**, The timing of fluid rupture and cellular rupture relative to egg release. Box, 25–75%; whiskers, 5–95%; centre, median; centre box, mean. Total *n* = 20, as shown in the top right of the graph. **d**, A model for the ovulation mechanism. (i) FSH drives follicle growth to the antral stage. Ovulation is initiated by a mid-cycle surge in LH. (ii) HA secretion drives cumulus and follicle expansion. The oocyte resumes meiosis I. (iii) Smooth muscle cells mediate follicle contraction. The follicle distends to form a stigma. (iv) The follicle wall thins and ruptures. The egg and cumulus mass are released from the follicle. Data in **a** and **c** are representative of seven independent biological repeats. Created with BioRender.com. Source data

We next asked how follicle rupture takes place. To address this, we performed confocal microscopy of the follicle wall during rupture and egg release. We additionally microinjected fluorescent dextran into the antrum to mark movement of the follicular fluid. Follicle rupture typically proceeded in three distinct steps (Fig. 5a and Supplementary Video 3). First, 63.9 ± 29.4 (mean ± s.d.) minutes before egg release, the follicles underwent fluid rupture, in which the fluorescent dextran leaked out of the follicle by penetrating between the cells of the follicle wall (Fig. 5a, step 1—fluid rupture; Fig. 5c). Second, 20.5 ± 13.2 (mean ± s.d.) minutes before egg release, the follicles underwent cellular rupture, in which the cumulus cells burst through a visible gap in the follicle wall (Fig. 5a, step 2—cellular rupture; Fig. 5c). During cellular rupture, the cumulus cells were contained within the cumulus matrix, which was marked as a clear mass by its separation from the fluorescent dextran-containing follicular fluid (Fig. 5a). Consistent with this, we frequently observed HA, a component of the cumulus matrix, moving ahead of the oocyte in cryosectioned follicles collected at 12 h post hCG addition (Fig. 5b). Finally, the egg was released (Fig. 5a; step 3—egg release). The temporal separation between fluid rupture, cellular rupture and egg release has been previously documented in living rats^53^. We suggest that the stepwise nature of follicle rupture reflects a gradual increase in the size of the gaps in the follicle wall as it is stretched during stigma formation.

**Extended Data Fig. 9:**
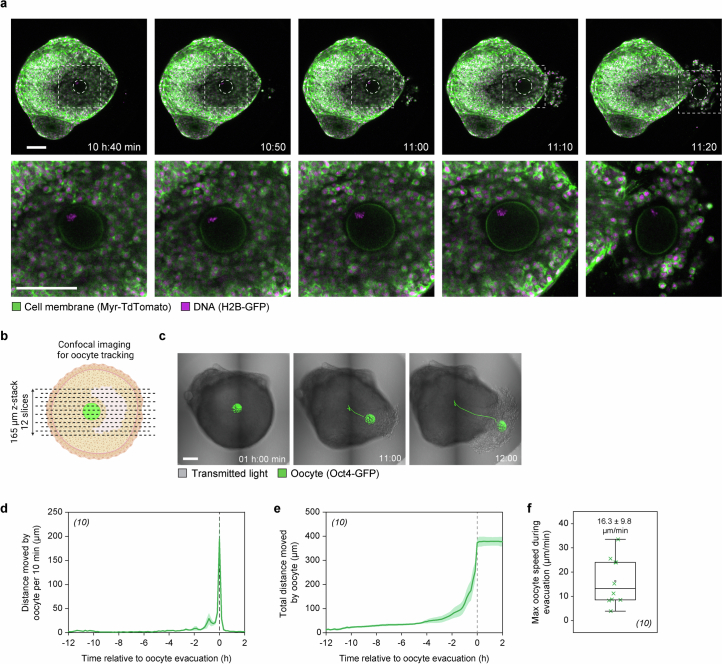
Rapid oocyte propulsion through the newly formed rupture site. (**a**) Representative confocal time course of oocyte movement out of the follicle. Dotted white square indicates region of inset; dotted white circle indicates oocyte outline; time relative to hCG addition; scale bar, 100 μm. (**b**) Schematic illustration showing the confocal imaging strategy for generating the 3D dataset for oocyte tracking. (**c**) Representative images of oocyte surface reconstruction through ovulation in isolated follicles expressing the oocyte marker Oct4-GFP. Green line shows the track of its movement in 3D during ovulation; green surface shows the oocyte. Time relative to hCG addition; scale bar, 100 μm. (**d**) Distance moved by the oocyte per 10 minute time interval during ovulation; *n* = 10. (**e**) Total distance moved by the oocyte; *n* = 10. (**f**) Plot showing maximum oocyte speed moving out of the follicle. Data in (c,d,e,f) is representative of 3 independent biological repeats. In panel (f): Box = 25-75%, whiskers = 5-95%, centre = median, centre box = mean. In panels (d,e): thick lines show the mean data, lighter shadows show the SEM. Created with BioRender.com. Source data

We next wanted to investigate the dynamics of oocyte movement during rupture. In our time-lapse data, we consistently observed that the oocytes remained largely stationary during follicle expansion and contraction, only moving to the follicle wall just before release (Extended Data Fig. 9a and Supplementary Video 1). To follow oocyte movement, we imaged follicles from transgenic mice expressing the oocyte marker Oct4–GFP, generated 3D surface reconstructions in Imaris and tracked the oocytes in three dimensions during ovulation (Extended Data Fig. 9b,c and Supplementary Video 4). The oocytes only started moving away from the follicle centre in the final hour before release, reaching their highest speed (16.3 ± 9.8 μm, mean ± s.d.) in the final 10–20 min as they surged through the rupture site (Extended Data Fig. 9d–f). This movement is in time with when we observe most follicles undergoing fluid and cellular rupture (Fig. 5c).

**Extended Data Fig. 10:**
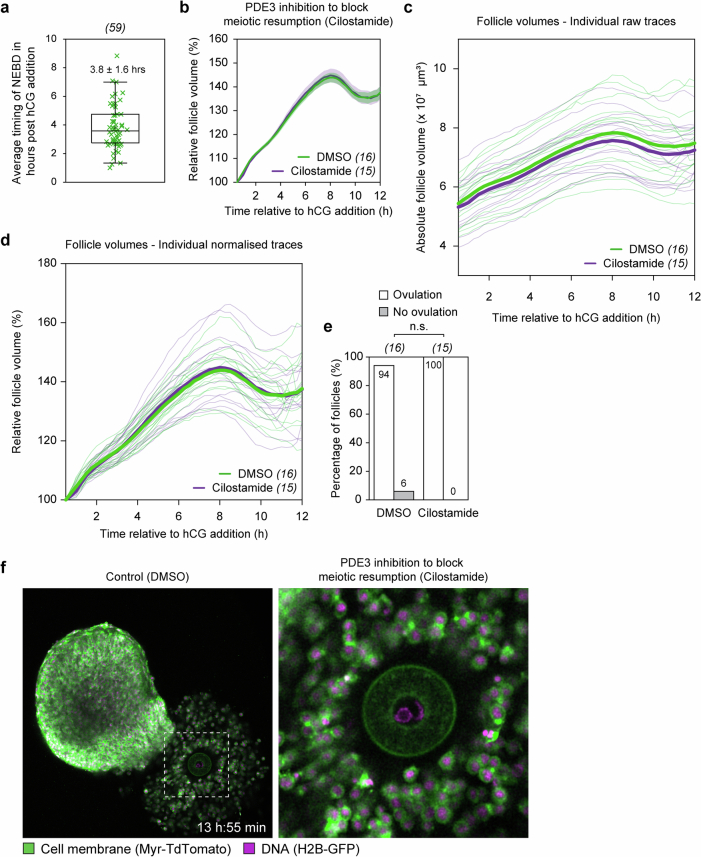
Meiotic resumption is not essential for ovulation. (**a**) Quantification of NEBD timing relative to hCG addition in control follicles. Only data from follicles that ovulated was included in this quantification; *n* = 59 from 14 independent biological repeats. Box = 25-75%, whiskers = 5-95%, centre = median, centre box = mean. Mean NEBD timing is displayed: 3.8 ± 1.6 h (s.d.) (**b**) Relative volume in control (DMSO) and cilostamide-treated follicles. (**c**) Graph showing absolute volume, non-normalised, in DMSO and cilostamide-treated follicles. (**d**) Graph showing volume normalised at 0.5 h post hCG addition in DMSO and cilostamide-treated follicles. (**e**) Quantification of ovulation rate in DMSO and cilostamide-treated follicles. Panels (b,c,d,e): total *n* = 31; individual groups: DMSO *n* = 16, cilostamide *n* = 15. P = 1. (**f**) Representative confocal images of ovulated GV oocyte in PDE3-inhibitor (cilostamide)-treated follicle. Data in panels (b,c,d,e,f) is representative of 2 independent biological repeats. In panel (e), chi-square test (two-sided) with Yates’s correction. ****P < 0.0001, ***P < 0. 001, **P < 0.01, n.s. non-significant, two-sided unpaired t-test. In panel (b): thick lines show the mean, lighter shadows show the SEM. In panels (c,d): thin traces show raw values from individual follicles; thick traces show the mean. Source data

As part of the ovulatory process, the oocyte matures into a fertilizable egg by progressing through the first meiotic division (Extended Data Fig. 10a). It has previously been shown that ovulation was unaffected in mice administered with a PDE3 inhibitor to block meiotic resumption in the oocyte^7^. When we treated follicles with the PDE3 inhibitor cilostamide^55^, the treated follicles both expanded and contracted normally (Extended Data Fig. 10b–d) and the ovulation rate was comparable to control follicles (94% ovulation in DMSO, 100% in cilostamide; Extended Data Fig. 10e). Strikingly, oocytes were ovulated with intact nuclei yet still surrounded by an expanded cumulus (Extended Data Fig. 10f). We thus confirm that neither ovulation nor cumulus expansion is dependent on meiotic resumption, consistent with previous findings^7^.

## Discussion

Live imaging studies of ovulation are impeded by the poor accessibility of the ovary, the narrow time window and the difficulty of predicting the future site of ovulation. As a result, ovulation has largely been studied in fixed tissues, limiting our understanding of the dynamics and progression of this essential stage of reproduction. Here, we have developed a system to visualize and probe the entire process of ovulation in isolated mouse follicles. This model system was validated by scRNA-seq, as well as immunofluorescence staining of key pathways controlling follicle expansion and contraction. Our work demonstrates that isolated ovarian follicles can act as single functional units, capable of responding to the ovulatory stimulus and undergoing ovulation with key morphological and transcriptional changes consistent with in vivo ovulation. We report that ovulation in isolated follicles consists of three phases that occur synchronously in all follicles, independent of variations in follicle size. In the first phase (follicle expansion), follicular cells secrete HA into the antrum surrounding the oocyte (Fig. 5d(i, ii)). This generates an osmotic gradient that drives fluid influx into the follicle, resulting in an increase in follicle volume. At 8 h post-hCG, the follicles enter the second phase (follicle contraction) in which smooth muscle cells around the follicle contract (Fig. 5d(iii)). We propose that an influx of fluid into the follicle driven by HA secretion, combined with inward pushing during contraction, leads to an increase in hydrostatic pressure within the follicle. When a threshold pressure is exceeded, the follicle distends towards the side with the thinnest follicle wall. This, in turn, causes a rapid stretching, thinning and eventual rupture of the wall, allowing the egg to be released (phase 3—rupture; Fig. 5d(iv)). During rupture, we observed fast movement of the oocyte from the follicle centre through the newly formed rupture site. We suggest that, during rupture, pressure is released from inside the follicle, leading to the rapid evacuation of the entire cumulus mass.

While we have directly demonstrated fluid influx into the follicle during expansion, the question remains as to the source of this fluid in vivo. We speculate that this may correspond to fluid movement from the local vasculature, as observed by labelled dextran in the blood that leaks into the follicle during intravital imaging in ovulating mice^17^.

Although isolated follicles successfully recapitulate the ovulatory cascade ex vivo, there are limitations to the system that should be considered when putting our observations back into the context of the whole ovary. First, we observed that isolated follicles expand isotropically. This is unlikely to be the case in the ovary, as antral follicles are positioned half exposed at the edge of the ovary. We therefore speculate that, in vivo, follicle expansion would be largely towards the outside, causing the follicle to protrude from the side of the ovary. Second, local vasoconstriction^17^ and protease activity^52^ have previously been shown to contribute to follicle rupture. We have shown that ovulation in isolated follicles is dependent on protease activity, specifically MMP2. However, we cannot investigate the role of vasoconstriction using our model system because it lacks functional vasculature. Other experimental systems will be more suitable to study this aspect of ovulation.

Our study includes an in-depth scRNA-seq dataset of carefully dissected ovulating follicles in vivo and ex vivo. During the submission of this manuscript, two studies using bulk RNA-seq^56^, and scRNA-seq and spatial transcriptomics^57^ of ovulating mouse ovaries were published. Along with our dataset, these studies will be valuable for the research community to discover novel pathways and cellular mechanisms driving ovulation.

In summary, we have established an experimental system that allows continuous visualization of events within the follicle during mammalian ovulation, at both the cellular and whole follicle level. The ex vivo ovulation system allows easy drug delivery and paves the way for mechanistic and systematic studies of ovulation. Our finding that the ovulatory phases are synchronous in isolated follicles demonstrates that, while an external cue is essential to initiate ovulation, the information for downstream events is self-contained within the follicle and independent of the rest of the ovary. Ovulation is therefore a remarkably robust process that requires carefully timed signalling events and complex coordination between the thousands of cells of the follicle.

## Methods

### Ethics

The maintenance and handling of all mice used in this study was performed in the Max Planck Institute for Multidisciplinary Sciences (MPI-NAT) animal facility according to international animal welfare rules (Federation for Laboratory Animal Science Associations guidelines and recommendations). Requirements of formal control of the German national authorities and funding organizations were satisfied, and the study received approval by the Niedersächsisches Landesamt für Verbraucherschutz und Lebensmittelsicherheit (33.19-42502-04-18/2746).

### Animals

Mouse (*Mus musculus*) strains used in this study were CAG-TAG (Srinivas lab, Oxford University; C57BL/6J background; 23–28 days of age), Oct4–GFP (Mann lab, Beckman Research Institute of the City of Hope, Duarte, CA; C57BL/6J background; 23–28 days of age) and C57BL/6J (23–28 days of age). All mice were female. Sex was determined by visual inspection of anal–genital distance of mice by a trained animal technician. All mice were kept in rooms with a constant temperature of 21 °C and humidity of 55%. The light–dark rhythm was 12:12 h, from 5:00 to 17:00. Health monitoring was carried out in accordance with Federation of European Laboratory Animal Science Associations recommendations with large annual examinations in January and smaller-scale examinations in May and September. For immunofluorescence and scRNA-seq experiments, 23–28-day-old C57BL/6J mice were superovulated by injection of 0.2 ml of 25 IU ml^−1^ pregnant mare serum gonadotropin (THP Medical Products, #hor-272-a) followed 48 h later by injection of 0.2 ml of 25 IU ml^−1^ hCG (Intervet, Ovogest 1000).

### Medium preparation

To prepare the basic culture and ovulation medium, 0.5 g of powdered Alpha-MEM (Thermo Fisher 12000014) and 0.11 g of sodium bicarbonate (final concentration 2.2 g l^−1^; Sigma-Aldrich, S5761) were dissolved in 50 ml of embryo-tested water (Sigma-Aldrich, W1503) and filter-sterilized using a 0.22 µm filter (Krackeler Scientific, SE2M228I04).

### Isolation and culture of mouse antral follicles

Our follicle isolation and culture protocol was adapted from previously established methods^11,15^. To obtain antral follicles, ovaries from 23–28-day-old mice were collected in Alpha-MEM + 25 mM HEPES (Thermo Fisher, 42360032) with 100 IU ml^−1^ penicillin–streptomycin (Gibco, 15140122), 5% foetal bovine serum (FBS; Gibco, 16000-044) and 30 ng ml^−1^ (1 nM) FSH (National Hormone and Peptide Programme, #NIDKK-oFSH-20 (ovine FSH)) (isolation medium). Ovarian antral follicles were isolated as previously described^15^ with minor modifications. Follicles of 300–500 µm diameter were dissected using 30 G needles in the collection medium detailed above, in 35 × 10 mm non-treated sterile culture dishes (CytoOne, CC7672-3340), then cultured in a 37 °C, 5% CO_2_, 20% O_2_ incubator for 24 h in media droplets on 0.4 µm polytetrafluoroethylene (PTFE) membrane cell culture inserts (Millicell, PICM03050) in six-well plates containing 1.6 ml Alpha-MEM (Thermo Fisher, 12000014) prepared with 2.2 g l^−1^ sodium bicarbonate (Sigma-Aldrich, S5761), supplemented with 100 IU ml^−1^ penicillin–streptomycin (Gibco, 15140122), 5% FBS (Gibco, 16000-044), 1× Insulin-Transferrin-Selenium (ITS-G; Gibco, 41400-045) and 30 ng ml^−1^ (1 nM) FSH (National Hormone and Peptide Programme, #NIDKK-oFSH-20 (ovine FSH)) (culture medium).

### Live imaging of ovulation in cultured follicles by confocal and two-photon microscopy

For ex vivo ovulation induction, follicles were transferred into Alpha-MEM (Thermo Fisher, 12000014) prepared with 2.2 g l^−1^ sodium bicarbonate (Sigma-Aldrich, S5761), supplemented with 100 IU ml^−1^ penicillin–streptomycin, 5% FBS (Gibco, 16000-044), 1× Insulin-Transferrin-Selenium (ITS-G; Gibco, 41400-045), 30 ng ml^−1^ (1 nM) FSH (National Hormone and Peptide Programme, #NIDKK-oFSH-20 (ovine FSH)), 8 µg ml^−1^ (5 IU ml^−1^) hCG (MSD Animal Health Ovogest 1,000 IU ml^−1^) and 5 ng ml^−1^ epidermal growth factor (EGF; Roche, 11376454001) (ovulation medium). The live imaging setup was constructed as follows. Standing feet were removed from the bottom of the 0.4 µm PTFE membrane cell culture inserts (Millicell PICM01050, 12 mm diameter) using scissors and attached to the bottom of a 35 mm glass-bottom dish (Ibidi, 81158) or a glass-bottom two-well slide (Ibidi, 80287) using imaging spacers (Grace Bio-Labs SecureSeal Imaging Spacers, 654002). Then, 1.3 ml of imaging medium was carefully pipetted into the dish and underneath the culture insert. Follicles were transferred into the re-equilibrated dish using glass capillaries with an inner diameter of 0.6 mm (Hilgenberg, 1411012), keeping them in individual droplets on the membrane, and the dishes were transferred to the microscope to start imaging as quickly as possible. The imaging chamber was supplied with 5.5% CO_2_ and heated such that the imaging medium measured at 37.5 °C. Wet tissues ensured sufficient humidity to prevent evaporation.

All microscopy was performed using ZEN Blue 2.3 (Zeiss). Images were acquired with LSM800 or LSM980 confocal laser scanning microscopes (Zeiss) with an environmental incubator box and a LD LCI Plan-Apochromat 25×/0.8 Imm Korr DIC M27 on the water-immersion setting in combination with Immersol W2010 immersion oil (Zeiss). To generate our two-dimensional datasets, we used tiling to cover the entire follicles and imaged two planes 30 µm apart at 10 min time intervals. For two-photon microscopy to generate our 3D datasets, follicles were imaged using a Zeiss LSM980 confocal laser scanning microscope with a Plan-Apochromat 20×/0.8 M27-Air and a MaiTai AX EHPDS two-photon laser (Spectra-Physics). For image acquisition, Myr–TdTomato was excited with the two-photon laser tuned to 1,000 nm at 30% power and detected using the GaAsP-BiG-non-descanned detector and a BP 570-610 nm bandpass filter. We imaged 27 slices through a *z*-stack of 260 µm with 4 tiled regions. Control and drug-treated groups were imaged on the same microscope. Care was taken to avoid phototoxicity and photobleaching. The step-by-step protocol for isolation, culture and imaging of mouse ovarian follicles is available on protocols.io^58^.

### Drug perturbation

Drugs used in this study include 4-MU (0.5 and 1 mM; Sigma-Aldrich, M1381), mifepristone (100 µM; Sigma-Aldrich, M8046), JKC-301 (10 µM; Sigma-Aldrich, SCP0141), FCCP (10 µM; Sigma-Aldrich, C2920), blebbistatin (−) and (+) (100 µM; Tocris, 1852 and 1853), Y-27632 (30 µM; Tocris, 1254) and SB-3CT (250 µM; Sigma-Aldrich, S1326). All drugs except mifepristone (resuspended in ethanol) were resuspended in DMSO (Sigma-Aldrich, D2650). Dextran for medium supplementation (25 and 50 mg ml^−1^; Sigma-Aldrich, D5376) was resuspended in the ovulation medium. For follicle expansion experiments (4-MU, dextran), follicles were incubated for 1.5 h in culture medium supplemented with the drug or DMSO, then transferred into the imaging setup. For contraction and rupture experiments (mifepristone, JKC-301, FCCP, blebbistatin, Y-27632 and SB-3CT), follicles were cultured in ovulation medium for 6 h before transfer into an equivalent medium containing the drug (or DMSO or ethanol) and live imaging was started immediately afterwards.

### Follicle volume measurements

To calculate follicle volume, images from 27 *z*-slices through the height of the follicle were segmented into (A) ‘follicle’ or (B) ‘background’ on the basis of the Myr–TdTomato signal using the pixel segmentation feature in Ilastik^59^. The segmented images were imported into Fiji/ImageJ^60^, and a mask was created with all holes in the signal filled. The images were 3D-reconstructed in Imaris, and volume measurements were extracted for all timepoints. In all main figure volume traces, follicle volumes were normalized to 100% at 30 min post hCG addition to represent how follicles expanded and contracted regardless of differences in starting volume. Raw and normalized individual traces for all control and drug-treated follicles are shown in Extended Data Figs. 4–6, 8 and 10.

### Follicle microinjection

Antral follicles were microinjected in isolation medium using a setup that has previously been described^61,62^. The protocol was adapted for injection of a high-molecular-weight (500,000 MW/500 kDa) fluorescently tagged dextran into the antrum of the follicle, instead of into the follicle-enclosed oocyte. The fluorescent dextran (Thermo Fisher, D7144) was labelled using the Alexa Fluor 647 Protein Labeling Kit (Thermo Fisher, A20173) according to the manufacturer’s instructions.

### Oocyte tracking

To track the oocyte, the imaging files were opened in Imaris. A surface was generated on the oocyte signal, and the movement of the centre of mass was tracked through all timepoints. Oocyte speed and total distance moved were extracted from Imaris and plotted.

### Tracking fluid influx with Alexa Fluor 647 Hydrazide dye

Antral follicles were isolated and cultured as described in the "Isolation and culture of mouse antral follicles" in Methods. Follicles were then transferred into imaging dishes containing (A) ovulation medium, (B) culture medium (without hCG) or (C) ovulation medium supplemented with 4-MU (1 mM; Sigma-Aldrich, M1381). All three medium types were additionally supplemented with Alexa Fluor 647 Hydrazide (10 µM; Thermo Fisher, A20502). Follicles were then transferred immediately to an LSM800 confocal laser scanning microscope (Zeiss), and images were acquired at 10 min intervals. Following acquisition, mean Alexa Fluor 647 Hydrazide fluorescence intensity measurements were made between mural granulosa cells, avoiding any antral regions, in Fiji/ImageJ.

### Immunofluorescence of cryosectioned follicles and ovaries

The staining protocol was adapted from previously published protocols^27,63^. Fresh ovarian follicles or ovaries from superovulated C57BL/6J mice were placed into plastic moulds (Leica HistoMold 6 × 8 mm, 14702218311) containing optimal cutting temperature (OCT) compound mounting medium for cryotomy (VWR Chemicals, 361603E), immediately frozen on dry ice and stored at −80 °C until sectioning. The frozen blocks were sectioned into 12 µm cryosections using a Cryostat (Leica, CM3050S), mounted onto microscopy slides (Thermo Fisher Scientific Menzel-Gläser Superfrost Plus, J1800AMNZ) and stored at −80 °C. Sections were fixed using a fixative containing 100 mM HEPES (pH 7.0, titrated with KOH), 50 mM EGTA (pH 7.0, titrated with KOH), 10 mM MgSO_4_, 4% methanol-free formaldehyde and 0.5% Triton X-100 in ddH_2_O at room temperature (RT) for 12 min, followed by three phosphate-buffered saline (PBS) washes for 5 min. Follicles were permeabilized in pre-chilled acetone (−20 °C) for 7 min, washed three times in PBS for 5 min each and blocked with 5% bovine serum albumin (BSA; Fisher Scientific, BP1605-100) in 1× PBT (PBS + 0.1% Tween) for 2 h at RT. For hyaluronan binding protein (HABP) staining, follicles were washed once with PBS + 5% BSA, stained using 1:50 dilution biotinylated HABP (b-HABP from Amsbio, AMS.HKD-BC41, 0.25 mg ml^−1^ stock concentration) in PBS with 5% BSA at 4 °C overnight. Sections were washed three times with PBS for 5 min, then incubated in PBS + 5% BSA with 1:500 Alexa Fluor 488-conjugated streptavidin (Thermo Fisher, S11223) for 2 h at RT. Sections were washed three times for 5 min in PBS + 5% BSA, stained with 100 µM Hoechst in PBS + 5% BSA for 5 min at RT, followed by three 5 min washes in PBS. For antibody incubations, blocked sections were stained with anti-smooth muscle actin (SMA) (rabbit polyclonal; Proteintech 55135-1-AP; 1:400; final concentration 1.5 μg ml^−1^) and anti-phospho-myosin light chain 2 (Ser19; mouse monoclonal; Cell Signaling #3675; 1:400) in blocking buffer (5% BSA in 1× PBT (PBS + 0.1% Tween)) overnight, washed using PBT, incubated with secondary antibodies (Alexa Fluor 647 chicken anti-mouse, Invitrogen A21200 or Alexa Fluor 488 donkey anti-rabbit, Invitrogen A31573, both at 1:500) for 1 h at RT, then washed for 1 h with PBT. All steps were carried out in a humid chamber. Samples were mounted using Prolong Glass Antifade Mounting Solution (Invitrogen ProLongGlass Antifade Mountant, P36984) and cover glasses (22 × 50 mm, 0.13–0.17 mm thick), and imaged within a week of staining using an LSM980 or LSM800 confocal laser scanning microscope (Zeiss) equipped with a multi-immersion LD LCI Plan-Apochromat 25×/0.8 Imm Korr DIC M27 (420852-9871-000) on the water-immersion setting or a C-Apochromat 40×/1.2 W Corr M27 (421767-9971-711) in combination with Immersol W2010 immersion oil (Zeiss).

### HABP staining intensity measurements

For HABP intensity quantifications, all samples were handled at the same time and using the same treatment throughout (two biological replicates and one technical replicate per biological replicate). All images were acquired with the same imaging settings on the same microscope. All measurements were done in Fiji/ImageJ^60^. The signal was only compared for the granulosa cell compartment by drawing custom regions of interest, excluding HABP signal in the outer layers of the follicle.

### Phosphorylated myosin light chain 2 occupancy analysis

For analysis of phosphorylated myosin light chain 2 occupancy on SMA, a mask was created using the SMA signal, and the intensity of both SMA and phosphorylated myosin light chain 2 signal was measured in the masked region. We then calculated the ratio between the intensities of SMA and phosphorylated myosin light chain 2 in the masked region.

### Generation of single-cell suspensions and scRNA-seq

For the in vivo dataset, follicles were mechanically isolated from ovaries of superovulated 23–28-day-old C57BL/6J mice at 0, 3, 6, 9 and 12 h post-hCG administration. For the ex vivo dataset, follicles from C57BL/6J mice were collected from the ex vivo culture system at 0, 3, 6, 9 and 12 h post-hCG addition. In both cases, three to five follicles were collected in low-binding 1.5 ml tubes (Eppendorf, VB-0285). To initiate the cell dissociation process, 1 ml of Collagenase IV (Gibco, 17104019) at 10 mg ml^−1^ was added to the samples. Samples were incubated for 30 min at 37 °C at 500 rpm, inverted every 5 min and pipetted with a wide-orifice low-retention tip (Mettler Toledo) every 10 min. After incubation, samples were pipetted ten times with a wide-orifice low-retention tip, pelleted at 400*g* for 5 min and incubated with Accumax (PAN-Biotech, P10-21200) for 3 min at RT with constant tube inversion, followed by termination of the reaction with 500 µl PBS supplemented with 10% FBS. The sample was further dissociated by pipetting using a wide-orifice low-retention tip and a normal-bore low-retention tip. The sample was strained twice through a 35 µm filter into a 1.5 ml low-bind tube, pelleted at 400*g* for 5 min at 4 °C, washed once with 500 µl PBS without Mg^2+^/Ca^2+^, resuspended in 100 µl PBS with 0.4% BSA using a wide-orifice low-retention tip and strained using Flowmi (Merck, 136800040). Viability and concentration were assessed using Trypan Blue with Countess II (Thermo Fisher Scientific, AMQAX1000).

The single-cell suspensions were processed using the 10x Genomics Chromium Single Cell System and the Chromium Single Cell 3′ v3 Reagent Kits following the manufacturer’s instructions. Around 12,000 cells per sample were loaded into the reaction well to recover around 7,000 cells per library. Following partitioning into gel bead-in-emulsion in the Chromium controller and downstream library preparation, samples were processed for paired-end sequencing in a NovaSeq 6000 by the Sequencing Core Facility, Max Planck Institute for Molecular Genetics, Berlin, Germany.

### Initial data processing and cluster annotation

Count matrices were converted to FASTQ files using the Cell Ranger software (version 3.0.2, 10x Genomics). Merging and quality check of the number of genes per cell, per cent mitochondrial content and number of total transcripts per cell were performed in Seurat v4.3.0.1^64^. Based on the simultaneous inspection of all parameters, the following quality check cut-offs were established: per cent mitochondrial genes ≤12.5%, per cent ribosomal genes ≤30%, number of unique genes detected >500, and total number of molecules >1,000 and <150,000. Doublet detection and removal were run on each sample separately using DoubletFinder v2.0.3^65^ with an assumed doublet formation rate of 5.6%. Overrepresented genes, such as Malat1, Gm42418 and AY036118, were filtered out to prevent technical bias. Normalization was performed with Scran v1.20.1^66^, and Harmony v1.2.0^67^ was used to integrate the datasets using the library preparation batch as the grouping variable.

Highly variable genes were identified using a mean variability plot with the following parameters: ‘mean.cutoff = (0.0125,3)’ and ‘dispersion.cutoff = (0.5,Inf)’. The best principal component analysis (PCA) dimension was chosen on the basis of either reaching over 90% cumulative variance with less than 5% individual variance, or if the difference in variance between successive components was greater than 0.1, whichever was lower. Uniform manifold approximation and projection (UMAP)^68^ was used on the Harmony embeddings for the downstream nonlinear dimensionality reduction. Clustering was performed using *K*-nearest neighbour graph^69^ and the Louvain algorithm^70^. The ideal cluster resolution was identified using Clustree v0.5.1^71^.

Cluster annotation was performed by inspection of the expression of known marker genes, temporal distribution of the cell types and examination of upregulated GO terms, as well as by performing direct comparisons of clusters to differentiate between closely related cell types.

### Differential gene expression and GO enrichment analyses

All differential gene expression analysis was performed using DESeq2 v1.32.0^72^. Pseudobulk counts were generated with Libra v1.0.0^73^. The following design was used for the direct comparison of timepoints: ‘~LPbatch + folliclecount + type + time’, where ‘LPbatch’ corresponds to the library preparation batch, ‘folliclecount’ to the number of follicles used in the sample, ‘type’ to the setup from which the follicles were isolated (either ex vivo or in vivo) and ‘time’ to the number of hours after hCG addition. Differential gene expression analysis was performed with the LRT test with ‘~LPbatch + folliclecount + type’ as the reduced variables. A gene was considered to be differentially expressed if the average absolute log_2_ fold-change of its expression (|avglog_2_FC|) was ≥0.25 and adjusted *P* value <0.05, and if it was expressed in at least 10% of the cells in the cluster. GO enrichment analyses were performed with the ‘enrichGO’ function from clusterProfiler v4.0.5^74^ and using genes with at least one unique molecular identifier count as the background.

For global comparison of the two setups (ex vivo versus in vivo) at each timepoint, the Wald test in DESeq2 v1.32.0^71^ with ‘~type’ as the design was used. Only genes that did not yield ‘NA’ *P* values (that is, genes with more than zero counts that are not outliers and with high mean normalized counts) in the differential gene expression analysis were used for the analysis. A gene was considered to be upregulated in a setup if its |avglog_2_FC| ≥0.5 and adjusted *P* value <0.01. Area-proportional Venn diagrams were generated with VennDiagram v1.7.3^75^.

### Figure preparation

Microscopy images shown in figures were processed using a Gaussian filter with a sigma of 1.0 in Fiji/ImageJ. Plots were generated using OriginPro 2022 (64-bit) SR1 9.9.0.225 and Graphpad Prism 9.3.1. All schematic diagrams were created with BioRender.com. Figures were assembled using Adobe Illustrator 27.1.1.

### Statistics and reproducibility

Sample size was determined by the maximum number of follicles that fit on the imaging membrane. This was typically between 5 and 12 follicles each for controls and drug inhibitions, and 15–25 in total. No statistical methods were used to pre-determine sample sizes. Follicles were not used for live imaging if they were deemed unhealthy on the basis of both follicle and oocyte morphology. Each experimental condition reported in this study includes at least two experimental replicates. Some conditions consisted of three or more experimental replicates. Biological replicates are indicated in the manuscript alongside the data figures, in the figure legends, and/or in Methods. Where applicable, each experiment in this study contained internal controls to ensure that variability between experimental replicates would not bias the outcome of experiments. Most experiments used follicles collected from multiple mice pooled together before random assignment to control and treatment groups. Control (DMSO-treated) follicles were always cultured and handled alongside drug-treated follicles. Follicles belonging to each treatment condition were processed and analysed blindly to avoid bias. While researchers knew the treatments involved in experiments, analysis of acquired data was performed blindly to limit bias. Automated software was used in analysis where possible. For statistical analyses, *P* values derived from two-sided unpaired *t*-tests were calculated using OriginPro 2022 (64-bit) SR1 9.9.0.225; *P* values derived from chi-squared test (two-sided) with Yates’s correction were calculated manually. Data distribution was assumed to be normal, but this was not formally tested. All data points are shown on graphs in which statistical testing has been done.

### Reporting summary

Further information on research design is available in the Nature Portfolio Reporting Summary linked to this article.

## Online content

Any methods, additional references, Nature Portfolio reporting summaries, source data, extended data, supplementary information, acknowledgements, peer review information; details of author contributions and competing interests; and statements of data and code availability are available at 10.1038/s41556-024-01524-6.

## Supplementary information

Reporting Summary

Supplementary Video 1 Ovulation in an isolated ovarian follicle expressing a cell membrane (Myr–TdTomato; green) and a chromosome (H2B–GFP; magenta) marker. Confocal microscopy allows the detailed study of both cellular and oocyte movements inside the follicle (inset).

Supplementary Video 2 High-resolution imaging of ovulation in an isolated ovarian follicle expressing a cell membrane (Myr–TdTomato; green) and a chromosome (H2B–GFP; magenta) marker. This revealed very low levels of cell division in the follicle (yellow rings) during the expansion phase.

Supplementary Video 3 Ovulation in an isolated ovarian follicle expressing a cell membrane (Myr–TdTomato; green) and a chromosome (H2B–GFP; magenta) marker, additionally injected with fluorescent dextran into the follicular antrum to mark movement of the follicular fluid (red). This video highlights the three steps of follicle rupture: fluid rupture (I), cellular rupture (II) and egg release (III).

Supplementary Video 4 Video of ovulation in an isolated ovarian follicle expressing the oocyte marker Oct4–GFP. This allowed for 3D surface reconstructions of the oocytes to be generated (green), to track their movement in 3D during ovulation. Transmitted light is also shown.

## Source data

Source Data Fig. 1 Source data for Fig. 1.

Source Data Fig. 2 Source data for Fig. 2.

Source Data Fig. 3 Source data for Fig. 3.

Source Data Fig. 4 Source data for Fig. 4.

Source Data Fig. 5 Source data for Fig. 5.

Source Data Extended Data Fig. 2 Source data for Extended Data Fig. 2.

Source Data Extended Data Fig. 3 Source data for Extended Data Fig. 3.

Source Data Extended Data Fig. 4 Source data for Extended Data Fig. 4.

Source Data Extended Data Fig. 5 Source data for Extended Data Fig. 5.

Source Data Extended Data Fig. 6 Source data for Extended Data Fig. 6.

Source Data Extended Data Fig. 7 Source data for Extended Data Fig. 7.

Source Data Extended Data Fig. 8 Source data for Extended Data Fig. 8.

Source Data Extended Data Fig. 9 Source data for Extended Data Fig. 9.

Source Data Extended Data Fig. 10 Source data for Extended Data Fig. 10.

## Data Availability

Sequencing data that support the findings of this study have been deposited in the Gene Expression Omnibus (GEO)^76^ under accession code GSE255274. Source data are provided with this paper. All other data supporting the findings of this study are available from the corresponding author on reasonable request.

## Code availability

Scripts for data processing and visualization of scRNA-seq data are available from https://gitlab.gwdg.de/schuh-meiosis/ovulation/-/tree/main.
