# Supplementary material for: Ex vivo imaging reveals the spatiotemporal control of ovulation
Source: Nat Cell Biol. 2024 Oct 16;26(11):1997–2008. doi: 10.1038/s41556-024-01524-6 (PMC11567896; doi:10.1038/s41556-024-01524-6)
Supplement: Supplementary file 1 — Reporting Summary [file 41556_2024_1524_MOESM1_ESM.pdf]

nature portfolio

Corresponding author(s): Melina Schuh

Last updated by author(s): Jul 9, 2024

# Reporting Summary

Nature Portfolio wishes to improve the reproducibility of the work that we publish. This form provides structure for consistency and transparency in reporting. For further information on Nature Portfolio policies, see our Editorial Policies and the Editorial Policy Checklist.

## Statistics

For all statistical analyses, confirm that the following items are present in the figure legend, table legend, main text, or Methods section.

| n/a | Confirmed | |
|---|---|---|
| ☐ | ☒ | The exact sample size (*n*) for each experimental group/condition, given as a discrete number and unit of measurement |
| ☐ | ☒ | A statement on whether measurements were taken from distinct samples or whether the same sample was measured repeatedly |
| ☐ | ☒ | The statistical test(s) used AND whether they are one- or two-sided<br>*Only common tests should be described solely by name; describe more complex techniques in the Methods section.* |
| ☒ | ☐ | A description of all covariates tested |
| ☒ | ☐ | A description of any assumptions or corrections, such as tests of normality and adjustment for multiple comparisons |
| ☐ | ☒ | A full description of the statistical parameters including central tendency (e.g. means) or other basic estimates (e.g. regression coefficient) AND variation (e.g. standard deviation) or associated estimates of uncertainty (e.g. confidence intervals) |
| ☐ | ☒ | For null hypothesis testing, the test statistic (e.g. *F*, *t*, *r*) with confidence intervals, effect sizes, degrees of freedom and *P* value noted<br>*Give P values as exact values whenever suitable.* |
| ☒ | ☐ | For Bayesian analysis, information on the choice of priors and Markov chain Monte Carlo settings |
| ☒ | ☐ | For hierarchical and complex designs, identification of the appropriate level for tests and full reporting of outcomes |
| ☒ | ☐ | Estimates of effect sizes (e.g. Cohen's *d*, Pearson's *r*), indicating how they were calculated |

*Our web collection on statistics for biologists contains articles on many of the points above.*

## Software and code

Policy information about availability of computer code

| | |
|---|---|
| Data collection | In this study, code was not used for data collection. Software used for data collection was ZEN Blue 2.3 (Zeiss). |
| Data analysis | Software used in analysis include Fiji/Image J version 1.54b44, Imaris x64 9.3.0, Ilastik version 1.3.3, OriginPro 2022 (64-bit) SR1 9.9.0.225, Graphpad Prism 9.3.1., ZEN Blue 2.3 (Zeiss), Adobe Illustrator 27.1.1, Cell Ranger (version 3.0.2, 10X Genomics), Seurat v4.3.0.1, DoubletFinder v2.0.3, Scran v1.20.1, Harmony v1.2.0, Clustree v0.5.1, DESeq2 v1.32.0, Libra v1.0 and ClusterProfiler v4.0.5. Scripts for data processing and visualization of single-cell RNA sequencing data are available from the following link: https://gitlab.gwdg.de/schuh-meiosis/ovulation/-/tree/main. |

For manuscripts utilizing custom algorithms or software that are central to the research but not yet described in published literature, software must be made available to editors and reviewers. We strongly encourage code deposition in a community repository (e.g. GitHub). See the Nature Portfolio guidelines for submitting code & software for further information.

## Data

Policy information about availability of data

All manuscripts must include a data availability statement. This statement should provide the following information, where applicable:

- Accession codes, unique identifiers, or web links for publicly available datasets
- A description of any restrictions on data availability
- For clinical datasets or third party data, please ensure that the statement adheres to our policy

Sequencing data that support the findings of this study have been deposited in the Gene Expression Omnibus (GEO) under accession code GSE255274. Source data are provided with this study. All other data supporting the findings of this study are available from the corresponding author on reasonable request.

## Human research participants

Policy information about studies involving human research participants and Sex and Gender in Research.

| | |
|---|---|
| Reporting on sex and gender | Not applicable |
| Population characteristics | Not applicable |
| Recruitment | Not applicable |
| Ethics oversight | Not applicable |

Note that full information on the approval of the study protocol must also be provided in the manuscript.

# Field-specific reporting

Please select the one below that is the best fit for your research. If you are not sure, read the appropriate sections before making your selection.

☒ Life sciences ☐ Behavioural & social sciences ☐ Ecological, evolutionary & environmental sciences

For a reference copy of the document with all sections, see nature.com/documents/nr-reporting-summary-flat.pdf

# Life sciences study design

All studies must disclose on these points even when the disclosure is negative.

| | |
|---|---|
| Sample size | Sample size was determined by the maximum amount of isolated follicles that would fit on the imaging membrane. This was typically between 10-15 follicles per treatment group and 20-30 in total. |
| Data exclusions | Follicles were excluded if they were deemed to be unhealthy prior to the start of the experiment based on both follicle and oocyte morphology. |
| Replication | Each experimental condition reported in this study includes at least two experimental replicates. Some conditions consisted of three or more experimental replicates. Biological replicates are indicated in the manuscript alongside the data figures, in the figure legends, and/or in the methods section. |
| Randomization | Where applicable, each experiment in this study contained internal controls to ensure that variability between experimental replicates would not bias the outcome of experiments. Most experiments used follicles collected from multiple mice of the same age group to be pooled together before random assignment to control and treatment condition groups. In addition, control (DMSO-treated) follicles were cultured alongside and in an identical fashion to drug-treated follicles. Follicles belonging to each treatment condition were then processed and analysed randomly in parallel to avoid bias. |
| Blinding | Control (DMSO-treated) follicles were always cultured and handled alongside drug-treated follicles. Follicles belonging to each treatment condition were processed and analysed blindly to avoid bias. While researchers knew the treatments involved in experiments, analysis of acquired data was performed blindly to limit bias. Automated software was used in analysis where possible. |

# Reporting for specific materials, systems and methods

We require information from authors about some types of materials, experimental systems and methods used in many studies. Here, indicate whether each material, system or method listed is relevant to your study. If you are not sure if a list item applies to your research, read the appropriate section before selecting a response.

## Materials & experimental systems

| n/a | Involved in the study |
| --- | --- |
| ☐ | ☒ Antibodies |
| ☒ | ☐ Eukaryotic cell lines |
| ☒ | ☐ Palaeontology and archaeology |
| ☐ | ☒ Animals and other organisms |
| ☒ | ☐ Clinical data |
| ☒ | ☐ Dual use research of concern |

## Methods

| n/a | Involved in the study |
| --- | --- |
| ☒ | ☐ ChIP-seq |
| ☒ | ☐ Flow cytometry |
| ☒ | ☐ MRI-based neuroimaging |

# Antibodies

| | |
| --- | --- |
| Antibodies used | Biotinylated Hyaluronan binding protein (b-HABP from amsbio, AMS.HKD-BC41);<br>AlexaFluor 488-conjugated Streptavidin (Thermo Fisher, S11223);<br>Anti-smooth muscle actin (rb polyclonal; Proteintech 55135-1-AP);<br>Anti-Phospho-Myosin Light Chain 2 (Ser19; mouse monoclonal; Cell Signaling #3675);<br>Alexa Fluor 647 chicken anti-Mouse (Invitrogen A21200);<br>Alexa Fluor 488 donkey anti-Rabbit (Invitrogen A31573). |
| Validation | - Biotinylated Hyaluronan binding protein was validated in our work by comparing staining in control follicles with follicles treated with an inhibitor of hyaluronic acid synthase (4-MU). This probe has also been previously validated in mouse ovarian follicles by the lab of Anthony Day, Manchester University, as shown on https://www.amsbio.com/hyaluronic-acid-binding-protein/<br><br>- Anti-smooth muscle actin was validated in our work by time course staining in follicles through ovulation. The staining pattern obtained was consistent with previous patterns shown in whole ovaries (https://academic.oup.com/biolreprod/article/102/5/1080/5712137). This primary antibody has also been previously validated in multiple mouse tissues, as shown on https://www.thermofisher.com/antibody/product/Alpha-SMA-Antibody-Polyclonal/55135-1-AP<br><br>- Anti-Phospho-Myosin Light Chain 2 was validated in our work by comparing staining in control follicles with follicles treated with an inhibitor of Rho-associated kinase (ROCK; Y-27632). This primary antibody has also been previously validated in mouse embryos, as shown in https://www.nature.com/articles/s41586-020-2695-9 |

# Animals and other research organisms

Policy information about studies involving animals; ARRIVE guidelines recommended for reporting animal research, and Sex and Gender in Research

| | |
| --- | --- |
| Laboratory animals | Mouse (Mus musculus) strains used in this study were: CAG-TAG (Srinivas lab, Oxford University; C57BL/6J background; 23-28 days of age); Oct4-GFP (Schöler lab, MPI for Molecular Biomedicine, Münster; C57BL/6J background; 23-28 days of age); C57BL/6J (23-28 days of age). All mice were female. Sex was determined by visual inspection of anal-genital distance of mice by a trained animal technician. All mice were kept in rooms with constant temperature of 21°C and the humidity of 55%. The light/dark rhythm was 12:12 hours, from 05:00 to 17:00. Health monitoring was carried out in accordance with Federation of European Laboratory Animal Science Associations recommendations with large annual examinations in January and smaller scale in May and September. For immunofluorescence and single-cell RNA sequencing (scRNAseq) experiments, 23-28 day old C57BL/6J mice were superovulated by injection of 0.2 ml of 25 IU/ml pregnant mare serum gonadotropin (THP Medical Products, #hor-272-a) followed 48h later by injection of 0.2 ml of 25 IU/ml human chorionic gonadotropin (Intervet, Ovogest® 1000). |
| Wild animals | The study did not involve wild animals. |
| Reporting on sex | Findings only apply to the female sex. Sex was determined by visual inspection of anal-genital distance of mice by a trained animal technician. |
| Field-collected samples | The study did not involve samples collected from the field. |
| Ethics oversight | The maintenance and handling of all mice used in this study was performed in the MPI-NAT animal facility according to international animal welfare rules (Federation for Laboratory Animal Science Associations guidelines and recommendations). Requirements of formal control of the German national authorities and funding organizations were satisfied, and the study received approval by the Niedersächsisches Landesamt für Verbraucherschutz und Lebensmittelsicherheit (LAVES). |

Note that full information on the approval of the study protocol must also be provided in the manuscript.
